# Growth and elongation of axons through mechanical tension mediated by fluorescent-magnetic bifunctional Fe_3_O_4_·Rhodamine 6G@PDA superparticles

**DOI:** 10.1186/s12951-020-00621-6

**Published:** 2020-04-25

**Authors:** Yang Wang, Binxi Li, Hao Xu, Shulin Du, Ting Liu, Jingyan Ren, Jiayi Zhang, Hao Zhang, Yi Liu, Laijin Lu

**Affiliations:** 1 Department of Hand Surgery, The First Hospital of Jilin University, Changchun, 130021 Jilin People’s Republic of China; 2 State Key Laboratory of Supramolecular Structure and Materials, College of Chemistry, Jilin University, Changchun, 130012 Jilin People’s Republic of China; 3 Institute of Translational Medicine, The First Hospital of Jilin University, Changchun, 130021 Jilin People’s Republic of China; 4 Departments of Geriatrics, The First Hospital of Jilin University, Changchun, 130021 Jilin People’s Republic of China

**Keywords:** Fe_3_O_4_ superparticles, Magnetic nanoparticles, Magnetic field, Mechanical forces, Magnetic actuation, Axon regeneration, Gene expression profile

## Abstract

**Background:**

The primary strategy to repair peripheral nerve injuries is to bridge the lesions by promoting axon regeneration. Thus, the ability to direct and manipulate neuronal cell axon regeneration has been one of the top priorities in the field of neuroscience. A recent innovative approach for remotely guiding neuronal regeneration is to incorporate magnetic nanoparticles (MNPs) into cells and transfer the resulting MNP-loaded cells into a magnetically sensitive environment to respond to an external magnetic field. To realize this intention, the synthesis and preparation of ideal MNPs is an important challenge to overcome.

**Results:**

In this study, we designed and prepared novel fluorescent-magnetic bifunctional Fe_3_O_4_·Rhodamine 6G@polydopamine superparticles (FMSPs) as neural regeneration therapeutics. With the help of their excellent biocompatibility and ability to interact with neural cells, our in-house fabricated FMSPs can be endocytosed into cells, transported along the axons, and then aggregated in the growth cones. As a result, the mechanical forces generated by FMSPs can promote the growth and elongation of axons and stimulate gene expression associated with neuron growth under external magnetic fields.

**Conclusions:**

Our work demonstrates that FMSPs can be used as a novel stimulator to promote noninvasive neural regeneration through cell magnetic actuation.
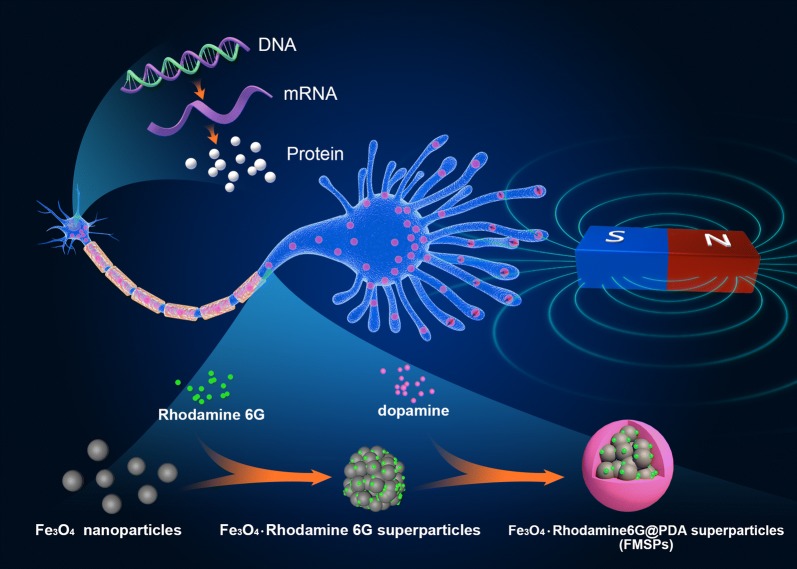

## Background

Nerve regeneration and neurological functional recovery are major issues in neuroscience regarding the treatment of peripheral nerve injuries [1]. Generally, neurons undergo a number of degenerative processes (Wallerian degeneration) after a peripheral nerve lesion. Benefiting from the intrinsic plasticity of the nervous system, functional reinnervation can be realized based on axonal sprouts penetrating and crossing the injured site from the proximal end to their distal target. Unfortunately, unsuccessful nerve regeneration often occurs in practice because peripheral nerve lesions may cause the degeneration of distal axons and scar infiltration at the site of the lesion, which not only poses a mechanical barrier to regenerating axons but also acts as a source of axon repellents [2]. As a result, detrimental changes occur, such as disorganized axonal sprouts, excess Schwann cell proliferation, and even painful neuromas. To date, the primary strategy to repair peripheral nerve injuries has been to bridge the lesions by promoting axon regeneration. Thus, the ability to direct and manipulate neuronal cell axon regeneration is one of the top priorities in the field of neuroscience.

Recently, the mechanical characteristics of the nervous system have received much attention since they can be used to direct the elongation and outgrowth of axons under external mechanical tension [3–5]. To accurately and noninvasively deliver external mechanical tension to axonal growth cones in vivo with controllable magnitude and duration, the application of magnetic nanoparticles (MNPs) such as Fe_3_O_4_ may be a promising option. Because of their magnetic properties, MNPs can be manipulated under inhomogeneous magnetic fields. For example, by incorporating MNPs into cells and placing the MNP-loaded cells in a magnetically sensitive environment in response to external magnetic field gradients, the remote guidance of neuronal regeneration can be achieved. It has been reported that iron oxide nanoparticles (NPs) can be exploited to promote nerve regeneration and provide guidance for regenerating axons [6–11]. However, because most of MNPs are larger than 20 nm, the particles are ferromagnetic rather than superparamagnetic. It is known that MNPs, including Fe_3_O_4_ or γ-Fe_2_O_3_, will become superparamagnetic rather than ferromagnetic when their diameters are less than 20 nm [12]. More importantly, since magnetic manipulation is an emerging research field, the genetic and molecular mechanisms that relate MNP stimuli and the biological behaviors of axon elongation are still unknown.

In this study, we demonstrate the preparation of novel fluorescent-magnetic bifunctional Fe_3_O_4_·Rhodamine 6G@polydopamine superparticles (FMSPs) for neural regeneration therapeutics. The as-prepared FMSPs possess ideal superparamagnetism since they are assembled from 5.8 nm Fe_3_O_4_ NPs. In contrast to individual Fe_3_O_4_ NPs, the assembled structure greatly accelerates the accumulation of Fe_3_O_4_ NPs in the cells, which is beneficial for providing stronger mechanical tension to manipulate the cells. In addition, the shell of polydopamine (PDA) endows our FMSPs with excellent biocompatibility and biodegradability. The manuscript first describes the characterization of the biocompatibility, cellular uptake mechanism, intracellular distribution and exocytosis of FMSPs. Then, the influence of the mechanical tension created by FMSPs under an external magnetic field on the growth and elongation of axons is examined. Third, the gene expression profile of neural cells under mechanical tension mediated by FMSPs is discussed based on mRNA transcriptome sequencing and bioinformatics analysis. The results indicate that our custom-made FMSPs can be endocytosed into neural cells via caveolae-mediated endocytosis and micropinocytosis by virtue of their excellent biocompatibility. By taking advantage of their high saturation magnetization and excellent superparamagnetism, the mechanical tension generated by FMSPs can promote the growth and elongation of axons and stimulate gene expression associated with neuron growth under external magnetic fields.

## Methods

### Materials and animal care

Bovine serum albumin (BSA), fetal bovine serum (FBS), Dulbecco’s modified Eagle medium (DMEM), neurobasal medium, NGF, penicillin/streptomycin, trypsin–EDTA, CellLight Golgi-RFP, MitoTracker Red, ER-Tracker Red, LysoTracker Red, Cytochalasin D (Cyto D), tubulin beta rabbit polyclonal antibody, goat anti-rabbit IgG secondary antibody, and antifade mountant with DAPI and Hoechst nucleic acid stains were obtained from Life Technologies Corporation (29851 Willow Creek Road, Eugene OR 97402, USA). Methyl-β-cyclodextrin (MβCD), genistein, chlorpromazine, amiloride, 2-deoxy-d-glucose (2-DG), and Cell Counting Kit-8 (CCK8) were purchased from Sigma-Aldrich (3050 Spruce Street, Saint Louis, MO 63103 USA). Rabbit anti-Cdh11, rabbit anti-Csf1r, mouse monoclonal anti-β-actin and anti-mouse antibodies were obtained from Santa Cruz Biotechnology (Santa Cruz, USA). The rabbit anti-Ppp1r1c antibody was obtained from Affinity (USA). The 35 mm imaging ibidi petri dishes (ibidi, 80156) were obtained from ibidi GmbH (Am Klopferspitz 19, 82152 Martinsried, Germany), and the two-compartment microfluidic chambers were purchased from Xona Microfluidics (Temecula, CA 92590, USA).

All animal procedures were performed in accordance with the Guidelines for Care and Use of Laboratory Animals of Jilin University and approved by the Animal Ethics Committee of Jilin University. All efforts were made to minimize the number of animals used and their suffering.

### Synthesis and characterization of FMSPs

FMSPs were prepared as described in our previous work [13]. Briefly, oleic acid (OA)-capped Fe_3_O_4_ nanoparticles (NPs) were synthesized following the thermal decomposition method [14]. The average diameter of the as-prepared Fe_3_O_4_ NPs was 5.8 nm. Subsequently, OA-capped Fe_3_O_4_ NPs dispersed in toluene and water containing sodium dodecyl sulfate (SDS) and Rhodamine 6G were mixed to form an oil-in-water (O/W) microemulsion. Fe_3_O_4_ superparticles (SPs) with an average diameter of 50 nm were produced after the evaporation of toluene [15, 16]. Finally, polydopamine (PDA) was coated on the surface of Fe_3_O_4_·Rhodamine 6G SPs via the oxidation polymerization of dopamine monomers under alkaline conditions [17].

The average size, distribution, and morphology of FMSPs were studied by high-resolution transmission electron microscopy (HRTEM) using a Hitachi H-800 electron microscope at an acceleration voltage of 200 kV with a CCD camera. The hydrodynamic size and zeta potential of the FMSPs were measured through dynamic light scattering (DLS) by a Zetasizer Nano-ZS (Malvern Instruments). Magnetic property characterization of the samples was performed at 300 K in a superconducting quantum interference device (SQUID) magnetometer (MPMS-XL, Quantum Design, Inc., San Diego, CA). Fluorescence spectroscopy was performed with a Shimadzu RF-5301 PC spectrophotometer.

### Cell and cell culture

To study the influence of magnetic manipulation on cellular behavior in neural cells, two neural cell types, rat adrenal pheochromocytoma cells (PC12, derived from a neuroendocrine tumor of the sympathetic nervous system, which is often used as a neuronal cell model) and primary rat dorsal root ganglia (DRG) neurons, were cultured. The PC12 cells used in this work were purchased from the Cell Bank of the Chinese Academy of Sciences (Shanghai, China) and cultured in DMEM supplemented with 10% FBS and 1% antibiotics (100 U/ml penicillin and 100 μg/ml streptomycin) [18, 19]. Primary DRG neurons were isolated from Sprague–Dawley rats (age 1–3 days) as described previously [20, 21] and maintained in neurobasal medium supplemented with 2% B27, 2 mM l-glutamine, 0.5% antibiotics (penicillin/streptomycin) and 50 ng/ml NGF. All cultures were conducted in an incubator at 37 °C in a humidified atmosphere with 5% CO_2_. The details of primary DRG neuron extraction are presented in Additional file 1.

PC12 cells were used for cellular behavior studies of the internalization of FMSPs, morphology analysis and examination of the magnetically guided outgrowth of neurites in directional orientation. DRG neurons were used as a model for examining magnetic tension achieved via FMSP interactions that stimulates the growth and elongation of axons at the single-cell level.

### Cytotoxicity assay

The CCK8 assay was used to examine the cytotoxicity of FMSPs. PC12 cells were seeded in 96-well plates at 1 × 10^4^ cells per well and incubated for 24 h. Different concentrations of FMSPs (0, 20, 50, 80, 100, 200 μg/ml) were added and incubated for another 24 h. Afterwards, the medium in each well was replaced with 90 μl of serum-free medium and 10 μl of CCK8. After 4 h of incubation, the absorbance of each well was measured at 450 nm using a Synergy HT microplate reader (Bio-Tek, Winooski, VT, USA). The survival rate of cells without FMSPs in the control wells was assumed to be 100%. Cell viability was derived through the absorbance percentage relative to the control cells.

### Quantification of internal FMSPs per cell

The cells were seeded in 6-well plates (5 × 10^5^ cells per well) and incubated for 24 h. After that, the cells were treated with FMSPs (10 μg/ml) for 4 h and washed twice with cold PBS containing 1 mM deferoxamine to remove iron that was nonspecifically attached to the cell membranes rather than taken up into the cells. Then, the cells were collected and counted. The amount of iron inside the cells in the samples was measured by ICP-AES measurements with a PerkinElmer Optima 3300DV. Based on the number of cells in the samples, we can calculate the average mass of iron per cell. From the density (ρ, 5.17 g/cm^3^) and volume (*V,* quasi-spheres with an average diameter of 50 nm) of the Fe_3_O_4_ SP core of the FMSPs, we can calculate the mass of each Fe_3_O_4_ SP core and the mass of iron in each FMSP. The number of FMSPs in each cell (*nFMSPs cell*) can be obtained by dividing the mass of iron in each cell by the mass of iron in each FMSP.

### Immunofluorescence staining

For immunocytochemistry, after 4 h of FMSP incubation, PC12 cells were washed twice with PBS to remove free-floating FMSPs, fixed for 30 min with 4% paraformaldehyde, permeabilized with 0.1% Triton X-100 for 10 min, and blocked with 2% BSA for 1 h at room temperature. The cells were labeled with tubulin beta rabbit polyclonal antibody at 2 µg/ml in 0.1% BSA, incubated overnight at 4 °C, and then labeled with goat anti-rabbit IgG secondary antibody with Alexa Fluor 594 conjugate (Ex/Em: 590/617 nm), a dilution of 1:2000 for 2 h at room temperature. Nuclei were stained with DAPI (Ex/Em: 405/430–470 nm).

### Cellular uptake kinetics

To study the effect of FMSP concentration on cellular uptake, PC12 cells were incubated with FMSPs at concentrations ranging from 5 to 20 μg/ml (5, 10, 15, 20 μg/ml). To study the effect of incubation time on the internalization of FMSPs, PC12 cells were incubated with FMSPs for 1, 2, 3, 4, 5 and 6 h. PC12 cells were seeded and incubated in 35 mm glass-bottom dishes (5 × 10^5^ cells per dish) for fluorescence microscopy imaging or in 6-well plates (5 × 10^5^ cells per well) for fluorescence activated cell sorter (FACS) analysis. A fluorescence microscope (IX51, Olympus Corporation, Tokyo, Japan) was used to examine the time- and concentration-dependent uptake, and the intracellular fluorescence intensity of FMSPs was qualitatively observed. For the FACS analysis (FACSAria II, Becton, Dickinson and Company, NJ, USA), the fluorescence intensity in cells was quantitatively measured with laser excitation at 561 nm and emission filtered at 582 nm, with a 15 nm band width; 10,000 events were collected for each sample.

In a separate experiment, energy-dependent uptake was performed with low-temperature incubation and ATP depletion treatments. PC12 cells were precooled at 4 °C or pretreated with 2-DG (50 mM for 45 min, creating an ATP-depleted environment by interfering with carbohydrate metabolism) at 37 °C, and then FMSPs were added and incubated for another 4 h. The intracellular fluorescence intensity of FMSPs was detected using fluorescence microscopy and FACS.

### Cellular uptake pathways

Fluorescence microscopy, FACS and TEM were used to study the exact uptake mechanism of FMSPs. Different endocytosis inhibitors were used to determine which pathway was mainly involved in the uptake of FMSPs according to the description in a previous publication [22]. Two millimolar MβCD (depleting cholesterol from the cell membranes) and 50 μg/ml genistein (inhibitor of tyrosine protein kinase blocking the phosphorylation of caveolin-1) were used to inhibit caveolae-mediated endocytosis. Chlorpromazine (10 μg/ml, inhibiting clathrin-coated pit formation) was used to inhibit clathrin-mediated endocytosis. Aminoride (50 μM, interfering with membrane Na+/H+ ATPase) and Cyto D (5 μM, inhibiting F-actin polymerization) were used to suppress micropinocytosis. PC12 cells were pretreated with inhibitors for 45 min, after which FMSPs (10 μg/ml) were added and coincubated for an additional 4 h prior to fluorescence microscopy and FACS analysis. Cells not incubated with FMSPs served as the negative control group, and a group treated only with FMSPs was used as the positive control.

Finally, TEM (EP 5018/40/Tecnai Spirit Biotwin 120 kV, FEI Czech Republic s.r.o, Holland) was used to study the successive stages of FMSP internalization. PC12 cells were incubated with FMSPs (10 μg/ml) for 4 h. Subsequently, cell samples were harvested and observed using TEM operating at 120 kV. Details of the specimen preparation are presented in Additional file 1.

### Intracellular trafficking and distribution

To further determine the intracellular trafficking and distribution of FMSPs, we assessed the colocalization of FMSPs with cellular organelles by using confocal laser scanning microscopy (CLSM, FV3000, Olympus Corporation, Tokyo, Japan) images. We used cellular organelle-specific fluorescent probes, including LysoTracker Red (Ex/Em: 561/610–710 nm), Golgi-RFP (Ex/Em: 561/580–680 nm), ER-Tracker Red (Ex/Em: 561/610–710 nm) and MitoTracker Red (Ex/Em: 561/610–710 nm), to determine the intracellular distribution of FMSPs (Ex/Em: 488/500–580 nm) by assessing the colocalization of FMSPs with lysosomes, Golgi apparatus, endoplasmic reticulum, and mitochondria, respectively. The experimental process was performed according to the manufacturer’s instructions (http://www.thermofisher.com). The images were captured with a 60× oil immersion objective with 3.2× magnification. Twenty different visual fields of each sample were analyzed, and triplicate experiments were performed. The colocalization of FMSPs with cellular organelles was analyzed by image analysis software ImageJ (http://rsb.info.nih.gov/ij/). Subsequently, as an additional validation experiment, TEM was used to directly observe the distribution and localization of FMSPs in the cellular ultrastructure.

### Exocytosis of internalized FMSPs

The exocytosis of internalized FMSPs was studied using FACS. After 4 h of preincubation with FMSPs, the cells were washed with PBS three times and cultured in fresh culture medium without FMSPs. After further incubation for 2, 4, 8, 12 and 24 h, the cells were collected for FACS analysis to measure the intracellular mean fluorescence intensity.

### Magnetic field preparation and quantification of magnetic forces

**Fig. 1:**
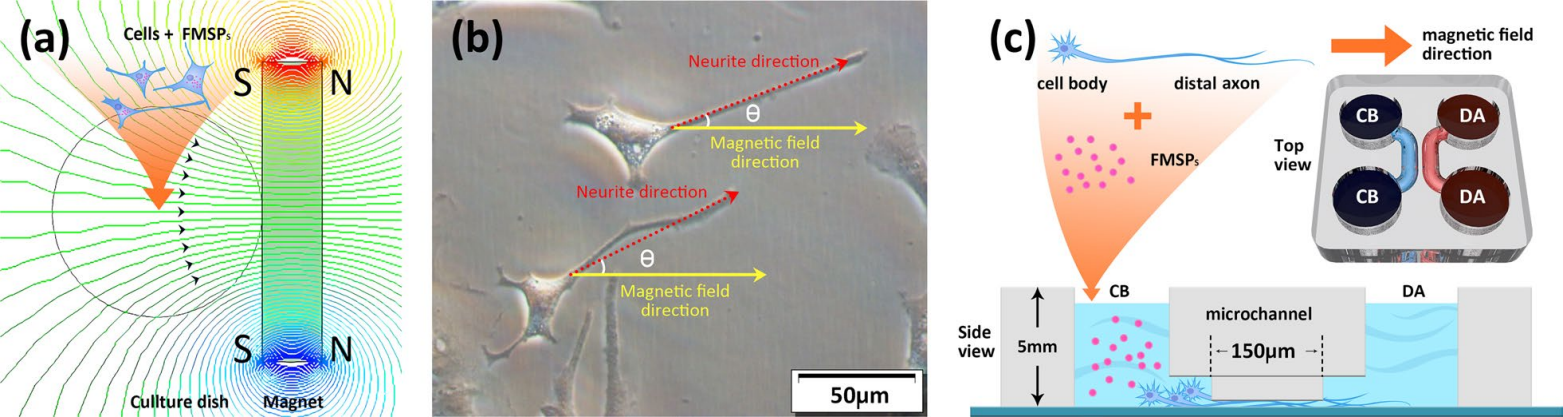
Magnetic applicator and experimental setup of neurite outgrowth. **a** Schematic illustration of the gradient magnetic field generated by the perpetual cuboid neodymium magnet applied to the cells at the center of the dish. **b** The angles θ between the long axis of the neurites (red dotted line) and the direction of the magnetic field (yellow solid line) (0 < θ < π). **c** Schematic illustration of a two-compartment microfluidic device for separating axons from cell bodies in DRG neuron culture

One perpetual cuboid neodymium magnet (NdFeB N48, cuboid side 50 mm × 30 mm × 10 mm) was applied to the right side of the culture dish to generate a gradient magnetic field to the cells at the center of the dish (Fig. 1a). The magnetic field was simulated by means of numerical field calculations using the software Comsol Multiphysics 4.3b (Comsol Multiphysics GmbH, Goettingen, Germany). A digital Gauss-meter (Scientific Equipment Roorkee, DGM-204) was used to measure the magnetic flux density induced by the setups of the neodymium magnet.

The exact explanation of the quantification of magnetic forces is as described in Refs. [6, 23]. A magnetic particle within a magnetic flux density gradient (∇*B*) experiences magnetic forces *F* directed toward regions with higher field density due to its magnetic momentum (*m*):

1 $$ F = \left( {m \cdot \nabla } \right)B. $$

In our experimental setup, the derivative of flux density *B*(T) along the magnetic field gradient inside the magnetic applicator is *dB/dr* (T/m). Superparamagnetic nanoparticles in gradient magnetic fields exert force due to a combination of parameters. As we find a value of FMSP saturation magnetization *M*_*s*_, the density *ρ* and volume *V*, we can assume the net force *F*_*FMSP*_ of FMSP:

2 $$ F_{FMSP} = m_{s} \frac{dB}{dr} = \rho VM_{s} \frac{dB}{dr}. $$

The mass of iron taken up by PC12 cells was measured, and the number of FMSPs per cell (*nFMSPs cell*) was calculated. A single cell will thus be subject to a force *F*_*cell*_ given by *F*_*FMSP*_ multiplied by the number of FMSPs in the cell:

3 $$ F_{cell} = n_{cell}^{FMSPs} \cdot F_{FMSP} . $$

### Neurite oriented growth assay

PC12 cells were used to examine the magnetically guided outgrowth of neurites in directional orientation. PC12 cells were seeded in ibidi petri dishes. After 24 h of seeding, cells were treated with 10 μg/ml FMSPs and incubated again for 4 h to allow the FMSPs to interact with cells. The dishes were then placed inside the magnetic applicator. After 24 h, the outgrowth of neurites was induced under an external magnetic force, and the angle θ between the long axis of a neurite, the direction of the magnetic field (Fig. 1b), and the neurite length were measured. Neurite orientation was quantified as an orientation index (Oi), which was defined as *Oi* = *cos*(θ), with 0 < θ < π (when θ is 0, neurites with their long axis parallel to the magnetic field vector will have an Oi of + 1, while when θ is π, neurites opposite to the magnetic field vector will have an Oi of − 1. Randomly oriented neurites will have an average Oi of 0).

Four experimental groups were tested: (1) the treatment group, cells treated with both FMSPs and magnetic field (FMSPs^+^, M^+^), (2) the FMSP control group, treated with FMSPs and no magnetic field (FMSPs^+^, M^−^), (3) the magnetic field control group, without FMSPs and treated with a magnetic field (FMSPs^−^, M^+^), and (4) the blank control group, without FMSPs and with a null magnetic field (FMSPs^−^, M^−^). Experiments were performed in triplicate. For each experiment, 625 pictures were acquired under microscopic high-power fields (20× objective with 2.5× zoom) at the centers of the dishes. For each picture, all of the neurites were measured. Analysis was performed using ImageJ software.

### Velocity measurement of DRG axonal elongation

To determine whether external magnetic force stimulation affects growth cone motility and the rates of axonal elongation in neurons, we used two-compartment microfluidic chambers for DRG neuronal culture. Previous studies have demonstrated that microfluidic chambers composed of two compartments (cell-body and distal-axon compartments) interconnected by microchannels (whose height is designed to be much smaller than the size of the cell body) can be used to separate axons from their cell bodies (Fig. 1c) [21, 24–27].

Neurons were initially plated in the left cell-body (CB) compartments, and cells extend long axons (~ 1 μm diameter) that are smaller than the microchannels and grow across the microchannels into the right distal-axon (DA) compartments after a few days. The growth and extension of axons from CB compartments to DA compartments via microchannels can be used to simulate the process of bridging nerve defects by axons in vivo. We used time-lapse imaging to monitor the dynamic process of axon growth continuously, including the characterization and quantification of the underlying behavior of individual axons and growth cones. During time-lapse recording, cultures were recorded at 2.5 min/frame with differential interference contrast (DIC) optics (20× objective with 2.5× zoom). The time for the extending axons to grow across 150-µm-long microchannels was recorded. The average elongation rates of axons were then constructed from each individual axon growth trajectory. In this study, the average speed of axons crossing through the microchannels instead of their directionless extension is measured as the axonal elongation rate. Experiments were carried out in triplicate, and more than 40 trajectories from a single axon were collected and measured in each experiment.

### mRNA transcriptome sequencing and bioinformatics analysis

To further determine the effects of mechanical signals mediated by FMSPs on the gene expression profile in neural cells, we also performed mRNA transcriptome sequencing and bioinformatics analysis on cell samples from different experimental groups. Experiments were carried out in triplicate.

RNA extraction, cDNA library establishment and Illumina sequencing: Total RNA was isolated from cultured sample cells using TRIzol reagent (Invitrogen, Carlsbad, CA, USA), and the purity, concentration and integrity of RNA were checked and controlled. mRNA enrichment was achieved by the combination of oligo (dT)-attached magnetic beads and the poly A tail. The mRNA interrupted fragments were synthesized into a strand of cDNA with random hexamer primers, and then the buffer solution, dNTPs and DNA polymerase used to synthesize the second-strand cDNA. The purified cDNA was terminally repaired, ligated, and PCR amplified to obtain the final cDNA library. The Illumina HiSeqTM 2000 sequencing platform was used for sequencing. All sequence data were submitted to the NCBI database under the Project accession number PRJNA597946 (https://www.ncbi.nlm.nih.gov/sra/PRJNA597946). To ensure the accuracy of subsequent bioinformatics, the original sequencing data were first filtered to obtain high-quality clean data. Then, using Hisat2 software (version 2.1.0, https://ccb.jhu.edu/software/hisat2/index.shtml) [28], sequencing reads were aligned to the rat reference genome (*R. norvegicus*, UCSC rn6; https://ccb.jhu.edu/software/hisat2/faq.shtml) to obtain high-quality sequencing data. Moreover, featureCounts software [29] (version 1.6.0) was used to analyze the gene expression level. The details of the quality control of the RNA extraction and the processing of raw sequencing data are presented in Additional file 1.

Screening differentially expressed mRNA: The obtained raw data were standardized with DESeq2 software (version 1.18.1, http://www.bioconductor.org/packages/release/bioc/html/DESeq2.html) [30], followed by pairwise comparison using the Wald test in DESeq2. Differentially expressed mRNAs were identified as those with P-values < 0.05 and |logFC| > 1. Gene Ontology (GO) analysis was utilized to analyze the putative functions of key genes using clusterProfile in R (version 3.8.1, http://bioconductor.org/packages/release/bioc/html/clusterProfiler.html) [31]. Upregulated differentially expressed mRNAs were subjected to GO biological process functional annotation [32] and enrichment analysis. A P-value less than 0.05 was used as the cut-off for the differential gene expression of significantly enriched GO terms.

### Quantitative real-time PCR

Cell samples from different groups were washed three times with precooled PBS. Cell lysis was performed for total RNA extraction by adding 1 ml TRIzol reagent. The obtained RNA was reverse transcribed to prepare cDNA using PrimeScript RT Master MIX (Takara, Dalian, China), followed by PCR amplification. PCR was used to quantify differences in mRNA expression using the PowerUp SYBR™ Green Master Mix Kit (Waltham, MA, USA). GAPDH was applied as an internal control. The primers used in PCR were synthesized by Invitrogen (Shanghai, China), and the sequences are listed in Additional file 1: Table S2. PCR conditions were as follows: initial denaturation at 95 °C for 10 min; 95 °C for 10 s, 60 °C for 30 s, and 72 °C for 1 min, and amplification was performed for 40 cycles, followed by extension at 72 °C for 10 min. The multiple relationships of gene expression changes in different cells were calculated by using the 2^−ΔΔCt^ method. Each reaction was performed three times.

### Western blots

Cell samples from different groups were washed with PBS and subsequently resuspended in radioimmunoprecipitation assay (RIPA, Beyotime, Shanghai, China) lysis buffer. Cell lysates were then collected by centrifugation (12,000 rpm for 15 min at 4 °C). Total proteins were quantified using the Bicinchoninic Acid (BCA, Thermo, Scientific, California, USA) Protein Assay Kit. Proteins were separated by 10% sodium dodecyl sulfate-polyacrylamide gel electrophoresis (SDS-PAGE), followed by transfer onto polyvinylidene difluoride (PVDF) membranes (Millipore, Bedford, MA, USA). A solution of 5% nonfat dry milk was used to block the membranes for 1 h. Subsequently, the membranes were incubated with the primary antibodies rabbit anti-Cdh11 (1:10,000), rabbit anti-Csf1r (1:10,000), rabbit anti-Ppp1r1c (1:10,000), and mouse monoclonal anti-β-actin (1:10,000) overnight at 4 °C. After three washes in PBST buffer, membranes were incubated with anti-mouse antibody (1:5000) at 37 °C for 1 h and then washed three times in PBST. The special bands were visualized using an electrochemiluminescence (ECL) method (Millipore, Bedford, MA, USA). TanonImage software (Tanon Science & Technology, Shanghai, China) was used to conduct grayscale analysis for protein expression. Experiments were carried out in triplicate.

### Statistical analysis

Continuous variables with normal distribution, such as cell viability, fluorescence intensity of cells, and elongation rates of axons, were represented as the mean ± standard deviation (SD). The distributions of the variables of neurite orientation index (Oi) and neurite length were found to be nonnormal by the Kolmogorov–Smirnov test; therefore, their values were represented by the median and interquartile range (Q1–Q3). Statistical significance was calculated using either one-way ANOVA (no rejection of normality) or nonparametric Kruskal–Wallis ANOVA (normality rejected). All of the analyses were conducted with SPSS (version 18.0, Chicago, IL, USA), and P < 0.05 was considered to be statistically significant.

## Results and discussion

### Synthesis and characterization of FMSPs

**Fig. 2:**
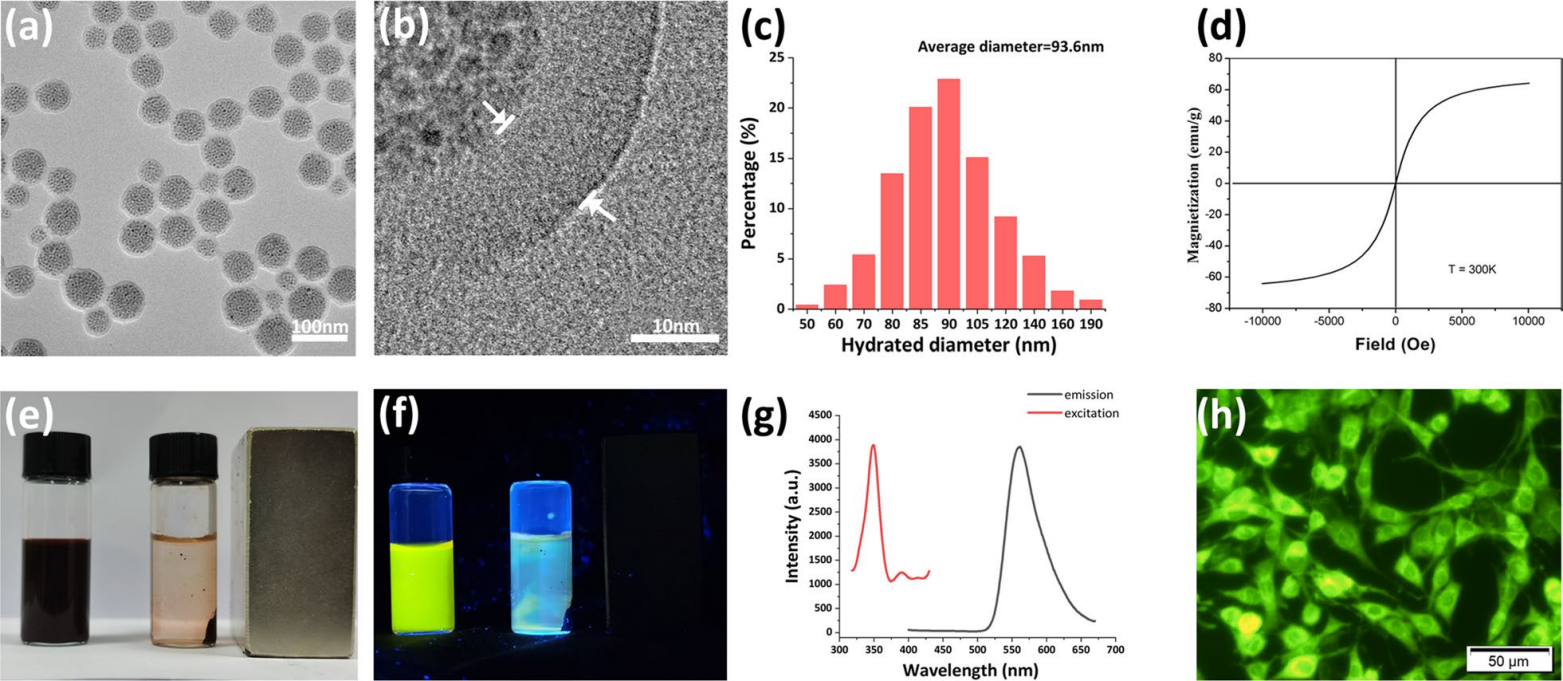
TEM (**a**) and HRTEM (**b**) images of FMSPs. **c** Size distribution of FMSPs. **d** Magnetic curves of FMSPs. Optical photograph (**e**) and fluorescent photograph (**f**) of FMSPs in the absence and presence of an external magnetic field. **g** PL emission and excitation spectra of FMSPs. **h** Fluorescence microscopy image of PC12 cells after incubation with FMSPs

The custom made Fe_3_O_4_·Rhodamine 6G@PDA SPs (FMSPs) used in this work are fabricated in-house by coating preassembled Fe_3_O_4_·Rhodamine 6G SPs with PDA. HRTEM images show that the FMSPs possess a uniform size with a core size of 50 nm and PDA shell of approximately 10 nm (Fig. 2a, b). DA is one of the most important neurotransmitters with widespread excitation in organisms, and the PDA shell can improve the biocompatibility, biodegradability and physiological stability of FMSPs. The hydrated diameter measured through DLS is 93.6 nm (Fig. 2c), which is larger than that measured in TEM images but still within the optimal range for uptake by nonphagocytic cells [33–35]. The polydispersity index (PDI) measured by DLS is 0.186, implying excellent dispersibility of our in-house fabricated FMSPs. Zeta potential measurement of FMSPs suspended in cell culture media reveals a weak negative charge of − 12.7 ± 2.3 mV, which is relatively favorable for their interactions with negatively charged cell membranes and subsequent uptake [10]. The magnetic curves of FMSPs are characterized by SQUID, and the saturation magnetization is 62 Am^2^/kg without any evident of remanence or coercivity at 300 K, suggesting that the FMSPs are superparamagnetic (Fig. 2d). In contrast to ferromagnetism, superparamagnetism allows magnetization to be saturated under an external magnetic field but randomized to zero upon suppressing the magnetic field [36]. As a result, using superparamagnetic nanomaterials can not only provide mechanical tensions in a magnetic field but also avoid undesired aggregation due to their null or negligible remnant magnetization in the absence of the magnetic field. Figure 2e illustrates the high magnetic responsivity of FMSPs toward an external magnetic field, which is the key parameter for maximizing the force transfer efficiency from the external magnetic field to the FMSP-loaded cells. The optical properties of FMSPs are investigated as well. As shown in Fig. 2g, FMSPs exhibit a green color emission at approximately 556 nm under 350 nm excitation. The photograph shown in Fig. 2f further indicates their bright green fluorescence excited by the hand-held UV lamp. PC12 cells after incubation with FMSPs exhibit a strong green fluorescence, which demonstrates that the FMSPs have an ideal capacity to label mammalian neural cells (Fig. 2h).

**Fig. 3:**
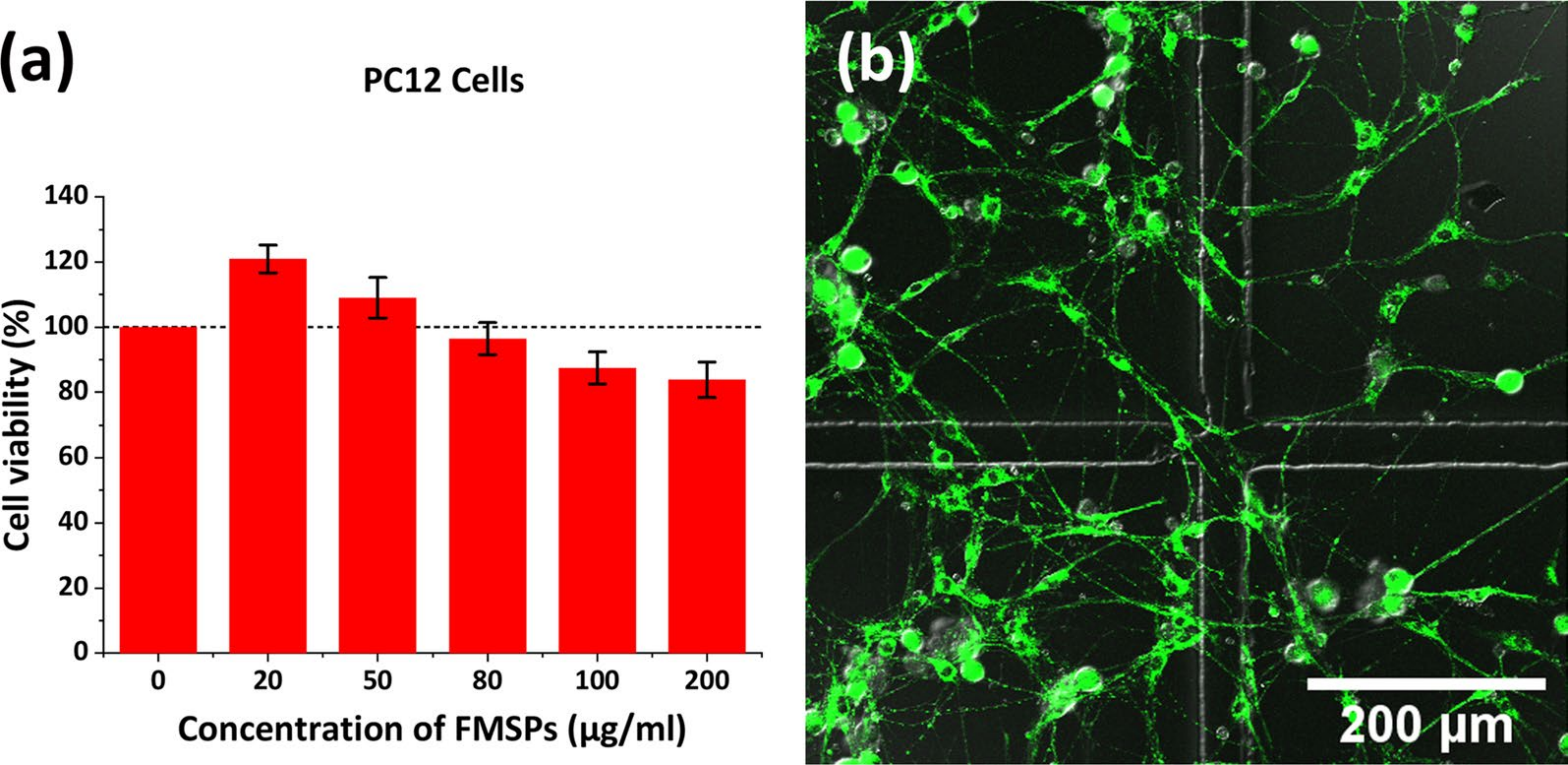
Cytotoxicity of FMSPs in PC12 cells. **a** Viability of PC12 cells after 24 h of coculture with different concentrations of FMSPs. **b** Outgrowth of FMSP-treated DRG neurons after incubation with FMSPs (10 μg/ml) for 72 h

Minimal cytotoxicity is essential for any biomedical application. The cytotoxicity of FMSPs was first evaluated by a standard CCK8 assay. After incubation with FMSPs for 24 h, the cell viability of PC12 cells remained above 80% at concentrations ranging from 20 to 200 μg/ml (Fig. 3a). Measurements of cell viability after 48 and 72 h in culture demonstrate a similar trend, implying that the uptake of FMSPs does not affect cell viability and replication rate. Additional file 2: Movie S1 shows that PC12 cells are able to maintain active mitotic proliferation and neurite outgrowth after the endocytosis of FMSPs. The amount of FMNP uptake by PC12 cells was calculated by ICP-AES. After incubation with FMSPs (10 μg/ml), the cells were able to incorporate quantities of Fe up to 6.90 ± 0.07 pg/cell, corresponding to ~ 3.73 ± 0.036 × 10^4^ FMSPs/cell. These data demonstrate that using 10 μg/ml as the working concentration not only guarantees extremely low cytotoxicity but also ensures sufficient FMSP uptake by cells. The low cytotoxicity of Fe_3_O_4_ is one of its great advantages compared with other MNPs [8, 23, 37, 38]. Our results are consistent with these studies and show that PDA-coated superparamagnetic Fe_3_O_4_·Rhodamine 6G SPs are basically noncytotoxic even at a high concentration of 200 μg/ml. Additionally, the effects of FMSP treatment on the normal differentiation capacity of DRG neuronal cells were examined. The results showed no significant change in the viability and differentiation capacity of DRG neurons even after incubation with FMSPs (10 μg/ml) for up to 72 h. DRG neurons treated with FMSPs retain their differentiation property, leading to neurite outgrowth and complex neuronal network formation (Fig. 3b and Additional file 3: Movie S2).

The idea of using mechanical tension mediated by MNPs to enhance the growth and elongation of axons provides a new approach for repairing neurological diseases and injuries. To this end, the design and preparation of ideal MNPs appears to be a challenge to overcome. Our results suggest that FMSPs are promising candidates as biocompatible magnetic agents for magnetically driven cell actuation.

### Cellular behaviors related to FMSP internalization

The uptake behavior of FMSPs by cells was further studied. CLSM images of cells in a single focal plane reveal high fluorescence within the cells, verifying the internalization of FMSPs into the cells (Additional file 1: Fig. S1). To assess the extent of FMSP internalization, the intracellular fluorescence intensity was measured by fluorescence microscopy and flow cytometry. As shown in Additional file 1: Fig. S2, the cellular uptake of FMSPs depends on the FMSP concentration in the medium. With increasing concentrations of FMSPs in the culture environment, the fluorescence intensity in cells and the intracellular amount of FMSPs increase. PC12 cells have a large uptake amount and high fluorescence levels even at low concentrations of FMSPs (10 μg/ml). Subsequently, the influence of incubation time on the uptake of FMSPs by PC12 cells was measured by fixing the FMSP concentration (10 μg/ml) but prolonging the incubation from 1 to 6 h. As a result, the fluorescence intensity increased rapidly within 1 h and reached a plateau after 2 h of incubation (Additional file 1: Fig. S3). The FACS analysis in Additional file 1: Fig. S3f, g shows that the uptake of FMSPs within 1 h is extremely high, a significant deceleration occurs between 1 and 2 h, and saturation is reached after 2 h.

Previous studies reported that the endocytosis process involved in NP cellular uptake is energy-dependent active transport [39–44]. To better evaluate the uptake kinetics, the cellular uptake of FMSPs at low temperature (4 °C) and in ATP-depleted environments (in the presence of 2-DG) was analyzed. Fluorescent images indicate that low temperature and ATP depletion can inhibit the cellular uptake of FMSPs (Additional file 1: Fig. S4a, d). As shown in Additional file 1: Fig. S4e, f, compared with the positive control group at 37 °C, PC12 cells at low temperature or in an ATP-depleted environment showed FMSP uptake decreases of 91.8 ± 0.3% (P < 0.05) and 65.6 ± 2.8% (P < 0.05), respectively. This result reveals the energy dependence of FMSP uptake.

**Fig. 4:**
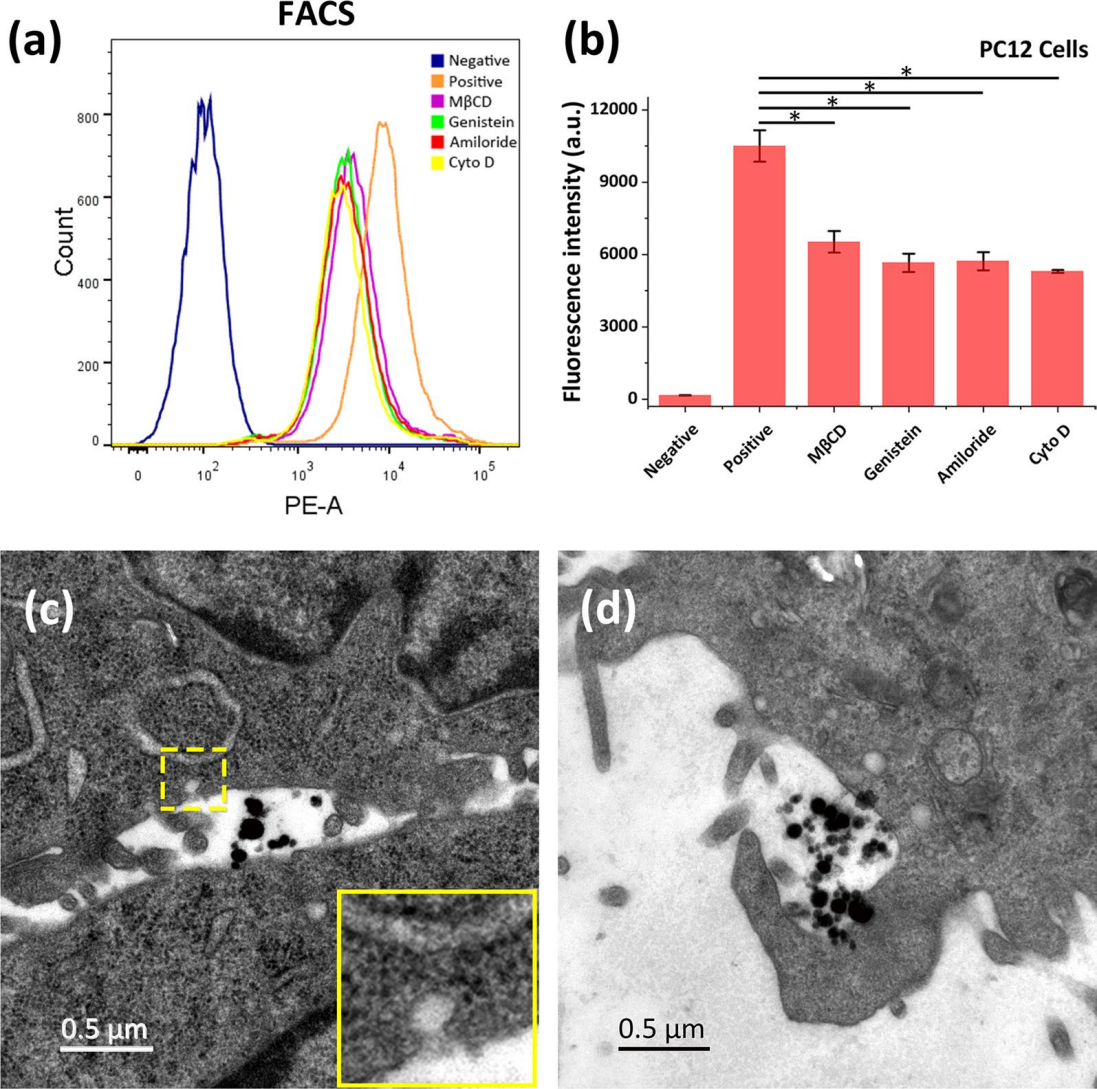
**a** FACS analysis for determining the endocytic pathways of FMSPs in PC12 cells. **b** Intracellular fluorescence intensity of PC12 cells in the presence of different endocytosis inhibitors. **c** TEM image of caveolae-mediated endocytosis. Insert is a magnified representative area (yellow box) showing the flask-shaped structure of caveolae (60–80 nm). **d** TEM image of micropinocytosis. The diameter and depth of the invagination structures conform to the characteristic features of micropinocytosis. *P < 0.05

The endocytosis of NPs in nonphagocytic cells generally includes caveolae-mediated endocytosis, clathrin-mediated endocytosis, and micropinocytosis [45, 46]. To elucidate the specific endocytic mechanism of FMSP uptake by cells, different endocytosis inhibitors were employed to identify the endocytic pathways involved in the uptake of FMSPs (Fig. 4a, b). MβCD and genistein were first utilized to inhibit caveolae-mediated endocytosis [47]. There was a significant decrease in FMSP uptake with inhibition rates of approximately 37.9 ± 4.3% (P < 0.05) and 46.1 ± 3.6% (P < 0.05) in PC12 cells. This result suggests that caveolae-mediated endocytosis is one pathway for FMSP uptake. Then, chlorpromazine was employed to inhibit clathrin-mediated endocytosis [47]. As a result, no obvious inhibition of FMSP uptake was found, indicating that clathrin-mediated endocytosis does not play an important role in the uptake of FMSPs (data not shown). Finally, amiloride and Cyto D were used to suppress micropinocytosis [48], and inhibition rates of approximately 45.5 ± 3.6% (P < 0.05) and 49.6 ± 0.6% (P < 0.05) were observed, indicating that micropinocytosis is a primary pathway for FMSP uptake. To obtain further detail, the trafficking of FMSPs into the cells was monitored by TEM analysis. Figure 4c shows dispersed FMSPs in the cell membrane appearing to enter the cells by caveolae-mediated endocytosis. Figure 4d shows clusters of FMSPs appearing to enter the cells by micropinocytosis. FMSPs were not observed in the nuclei, and no damage to the cytoplasmic organelles was found.

**Fig. 5:**
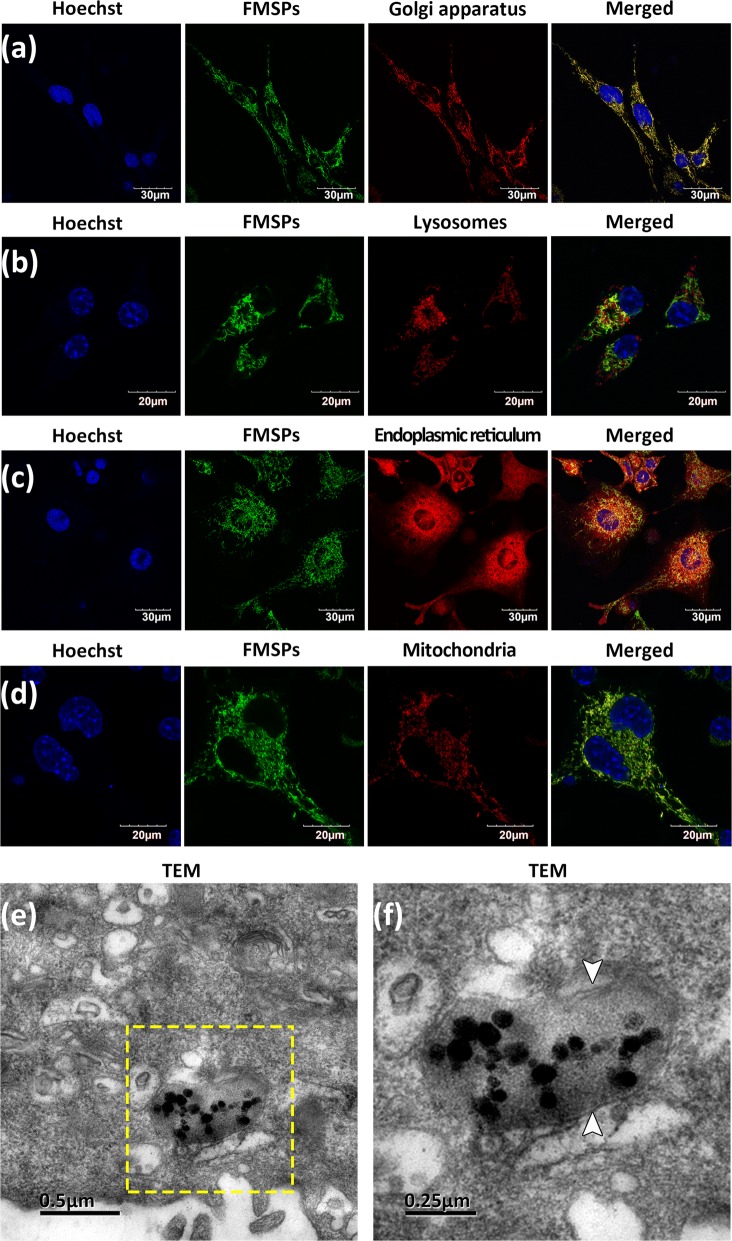
CLSM images of the colocalization of FMSPs with the Golgi apparatus (**a**), lysosomes (**b**), endoplasmic reticulum (**c**) and mitochondria (**d**). **e** TEM image of FMSPs distributed in mitochondria. **f** The enlarged local area of (**e**) demonstrates FMSPs with high electron density and the characteristic double-layer membrane structure (white arrows) of mitochondria

CLSM was further performed to identify the biodistribution of FMSPs within nerve cells. The intracellular distribution of FMSPs was evaluated via coincubation followed by costaining with various organelle-specific fluorescent probes (LysoTracker, ER-Tracker, Golgi-RFP, and MitoTracker) (Fig. 5a–d). Figure 5a, d show that internalized FMSPs were primarily distributed in the Golgi apparatus and mitochondria of PC12 cells. Accordingly, the Pearson correlation coefficients (Rr) were 0.656 ± 0.067 and 0.624 ± 0.026, respectively, suggesting good colocalization between FMSPs and these subcellular structures. Most importantly, our FMSPs could be observed not only in the cytoplasm but also in the growth cones of developing neurites. With the help of CLSM, tomographic scanning and time-lapse imaging are performed to clearly distinguish the intracellular distribution of FMSPs. Additional file 4: Movie S3 shows that FMSPs are transported bidirectionally within neurites and can be detected as puncta in the growth cones, neurites, and cell bodies.

To study the exocytosis of internalized FMSPs, cells were first preincubated with FMSPs for 4 h, then washed with PBS and incubated with fresh culture medium for 2, 4, 8, 12 and 24 h at 37 °C. Additional file 1: Figure S5 shows a significant decrease in intracellular FMSP fluorescence intensity with increasing incubation time. However, cells still maintained a high survival rate throughout the test period. The effective exocytosis of FMSPs further demonstrated the low cytotoxicity and high biocompatibility of FMSPs.

A comprehensive study of the neural cell behaviors involved in FMSP uptake is highly indispensable for assessing the biological features and subsequent biomedical applications of FMSPs. The findings derived from this study not only provide some details on neural cell behaviors involved in FMSP uptake but also lay the foundation for using the mechanical force mediated by FMSPs to guide the regeneration of axons.

### Magnetic mechanical forces induce axonal outgrowth

To prove that FMSPs can be exploited to manipulate the growth and development of neurites/axons under external magnetic fields, experiments were carried out inside a constant magnetic flux density gradient generated by one perpetual cuboid neodymium magnet, which provided approximately 6.0 T/m of magnetic field to the cells at the center of the dish along the direction of the magnetic field gradient (Additional file 1: Fig. S6). The custom made FMSPs used in this work had an average Fe_3_O_4_ core diameter of 50 nm (volume of approximately 6.54 × 10^4^ nm^3^) and a saturation magnetization of 62 Am^2^/kg. Since the density (ρ) of Fe_3_O_4_ is 5.17 g/cm^3^, a single FMSP was subjected to a force *F*_*FMSP*_, calculated to be ~ 1.15 × 10^−4^ pN (Eq. 2). The number of FMNPs taken up by PC12 cells was estimated to be ~ 3.73 × 10^4^ FMSPs per cell. Thus, the average magnetic force on a single cell *F*_*cell*_ was calculated to be ~ 4.29 ± 0.042 pN (Eq. 3).

**Fig. 6:**
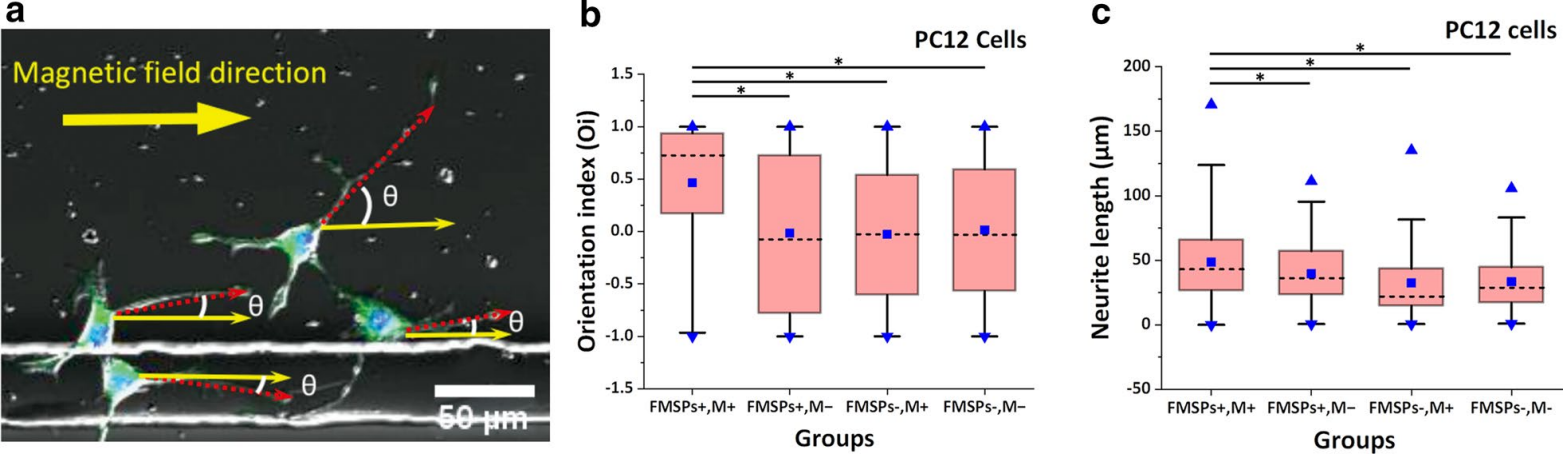
**a** Magnetic guidance outgrowth of PC12 cell neurites in directional orientation in the treatment group (FMSPs+, M+). The value of the orientation index (**b**) and neurite length (**c**) in the treatment group compared to the control groups. A total of 301, 238, 255 and 340 neurites were collected and measured in the four experimental groups, namely, the treatment group, FMSP control group, magnetic field control group and blank control group, respectively. The box plot shows the median (whiskers), interquartile ranges (boxes), and 5–95% percentiles (line). The square represents the mean value, and triangles represent the maximum and minimum. *P < 0.05

PC12 cells loaded with FMSPs were used to examine the effect of magnetic forces on the growth of neurites under an external magnetic field. The inclination angles θ between the long axis of the neurites and the line drawn parallel to the magnetic field were measured (Fig. 6a). Neurite orientation was quantified by introducing the concept of the orientation index (Oi). Figure 6a and Additional file 5: Movie S4 show that the neurites of PC12 cells treated with FMSPs (FMSPs^+^, M^+^) tended to be arranged in parallel with one another and to grow preferentially along the direction of the magnetic force when the magnetic field was applied. In contrast, the neurite growth directions for the control neurons appeared to be random with no preferred direction in the absence of magnetic stimulation. Furthermore, experimental evidence demonstrated that neither the FMSPs nor the magnetic field alone can influence the neurite growth direction. The value of Oi in the blank control group (FMSPs^−^, M^−^) was − 0.032 (− 0.571 to 0.604), which was not significantly different from that obtained when the magnetic field was applied (FMSPs^−^, M^+^; Oi = − 0.027, *P* = 0.493) or cells were treated with FMSPs (FMSPs^+^, M^−^; Oi = − 0.076, *P* = 0.580) alone (Fig. 6b). In contrast, Oi in the treatment group (FMSPs^+^, M^+^) was 0.726 (0.171–0.936), which was much higher than that in other groups (*P* < 0.05) (Fig. 6b). Concerning the length distribution of neurites along the magnetic force direction, a trend toward statistical significance (*P* < 0.05) was observed with increasing neurite length for cells treated with both FMSPs and the magnetic field (43.115 μm, 26.370–65.817 μm) compared to the control group (36.110 μm in FMSPs control group, 21.885 μm in magnetic field control group and 28.289 μm in blank control group, *P* < 0.05) (Fig. 6c).

**Fig. 7:**
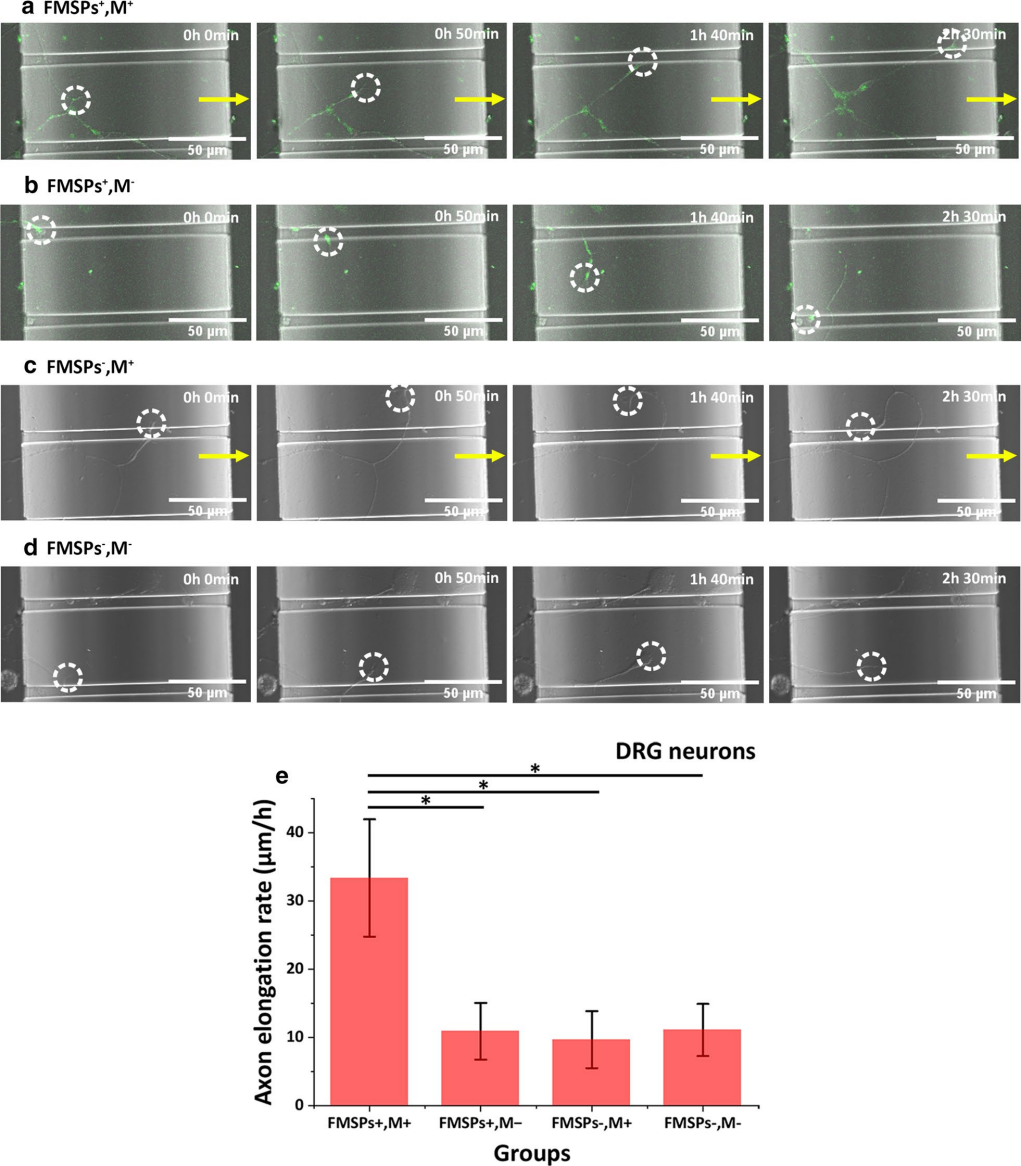
Time-lapse imaging of growth cone motility and axon elongation of DRG neurons in the **a** treatment group (FMSPs+, M+), **b** FMSP control group (FMSPs+, M−), **c** magnetic field control group (FMSPs−, M+) and **d** blank control group (FMSPs−, M−). **e** The average elongation rate of the axons in the treatment group compared to the control groups. A total of 173, 145, 154 and 138 axons were collected and measured in the four experimental groups, namely, the treatment group, FMSP control group, magnetic field control group and blank control group, respectively. The yellow arrows represent the direction of the magnetic field. The white circles highlight the growth cones. The images in **a**–**d** were captured at intervals of 50 min. *P < 0.05

Functional reinnervation after peripheral nerve injury is wholly dependent on the growth and extension of axons from the proximal end across the injured site until they reach their distal target. Therefore, from the perspective of promoting peripheral nerve regeneration and repair, the orderly and organized growth of axons in a particular direction is crucial. To determine the effects of nanomagnetic force stimulation on the growth cone motility and axon elongation rate, primary DRG neurons were cultured inside microfluidic chambers. The growth and extension of axons from cell-body (CB) compartments to distal-axon (DA) compartments via the microchannels was used to simulate the process of bridging nerve defects by axons in vivo, and the average speed of axons crossing the microchannels was measured as the elongation rate. In the treatment group (FMSPs^+^, M^+^), neurons were dissociated and incubated in medium enriched by FMSPs under an external magnetic field with a magnetic gradient. Figure 7a and Additional file 6: Movie S5 show that the magnetic force acting on the FMSP-bound axons DRG neurons allowed the growth cone to grow rapidly and directionally toward the distal-axon (DA) compartment (magnetic source) within a short time by crossing the microchannels. The growth of DRG neurons in a microfluidic chamber was monitored for more than 24 h by time-lapse imaging, and the average elongation rate of the axons was 33.4 ± 8.6 μm/h throughout the observation period. In the control groups (FMSPs^−^, M^−^; FMSPs ^+^, M^−^ and FMSPs^−^, M^+^) without nanomagnetic force stimulation, axons emerged from the neuron cell bodies spontaneously and developed in all directions without any preferred orientation (Fig. 7b–d and Additional file 7: Movie S6, Additional file 8: Movie S7, Additional file 9: Movie S8). This caused the axons to fail to pass through the microchannels quickly and efficiently, which explains why the rates of axon growth were decreased significantly in these control groups (11.1 ± 3.8 μm/h in the blank control group, *P* < 0.05; 10.9 ± 4.2 μm/h in the FMSP control group, *P* < 0.05 and 9.7 ± 4.2 μm/h in the magnetic field control group, *P* < 0.05) (Fig. 7e). Thus, it is reasonable to believe that the synergistic combination of FMSPs and an external magnetic field can improve the motility of the growth cone by aligning the direction of the magnetic force and accelerating the directional outgrowth of axons.

Axon elongation is the result of axonal framework stretches caused by tension from the growth cone under external chemical or physical stimulation [49]. More recently, mechanical forces in the range of several pN have been reported to influence the outgrowth process with the help of an MNP-based technique [6, 7, 10, 11, 50]. The experimental setups have significant effects on the measurement of the force applied to the cell since the value of this kind of force is too small. With superparamagnetic FMSPs, the magnetic force generated by the FMSPs can be controlled remotely and precisely by adjusting the intensity of the external gradient magnetic field. In our system, we demonstrate that internalized FMSPs in neural cells may generate a long-acting, low-order (~ 4.29 ± 0.042 pN) tension force, which is sufficient to physically direct oriented neurite outgrowth and axonal elongation. Based on previous works and our findings, we believe that precise control of the magnitude and duration of mechanical forces enables manipulation of the development of axons.

### Gene expression mediated by magnetic mechanical forces

**Fig. 8:**
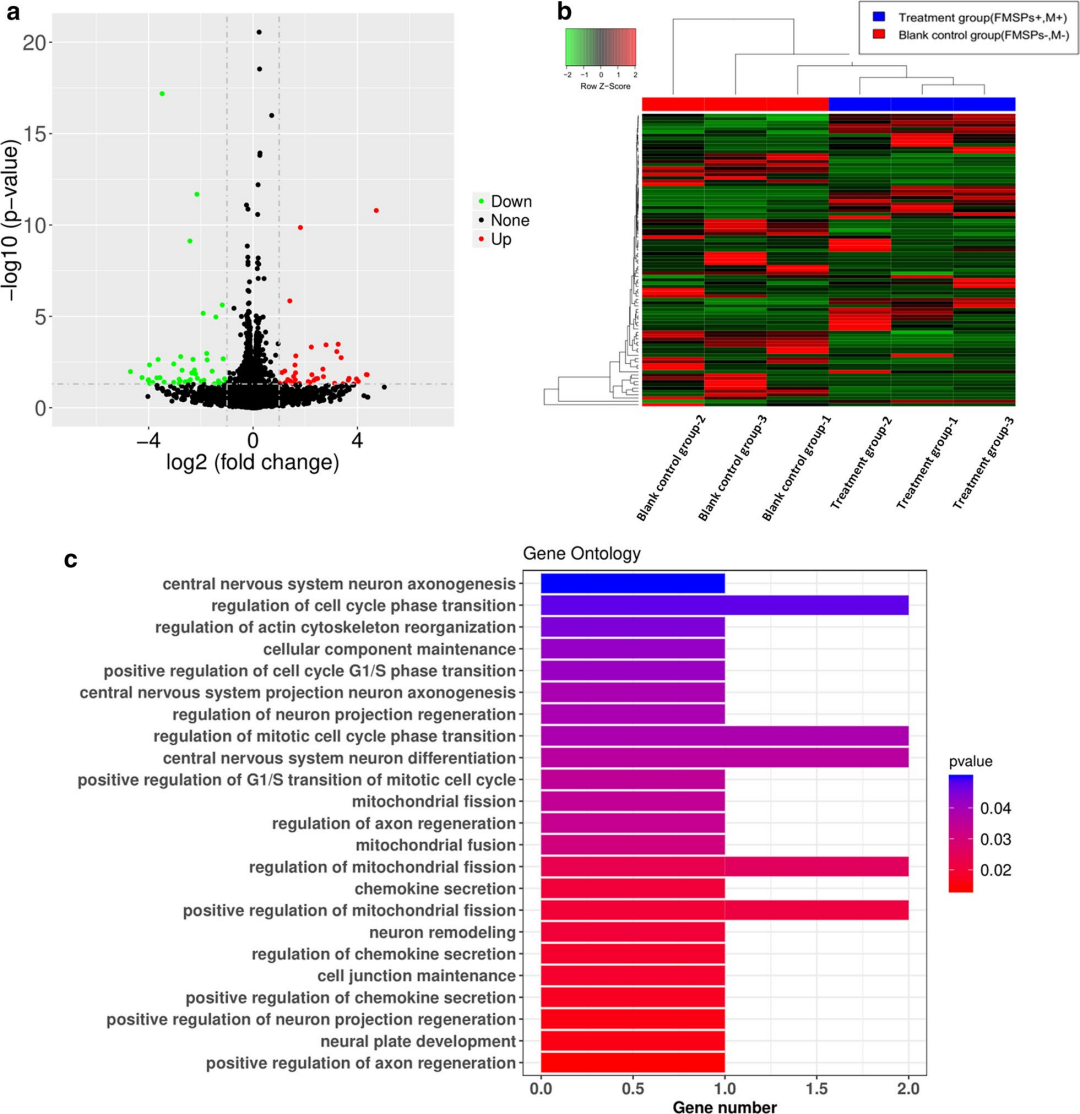
Screening differentially expressed mRNA. **a** The volcano plot of all differentially expressed mRNAs. Red and green dots represent up- and downregulated mRNAs, respectively. **b** Heat map visualization of the patterns of expression change for the significantly differentially expressed mRNAs between the treatment and blank control groups. **c** Gene Ontology enrichment analysis associated with axon growth

**Fig. 9:**
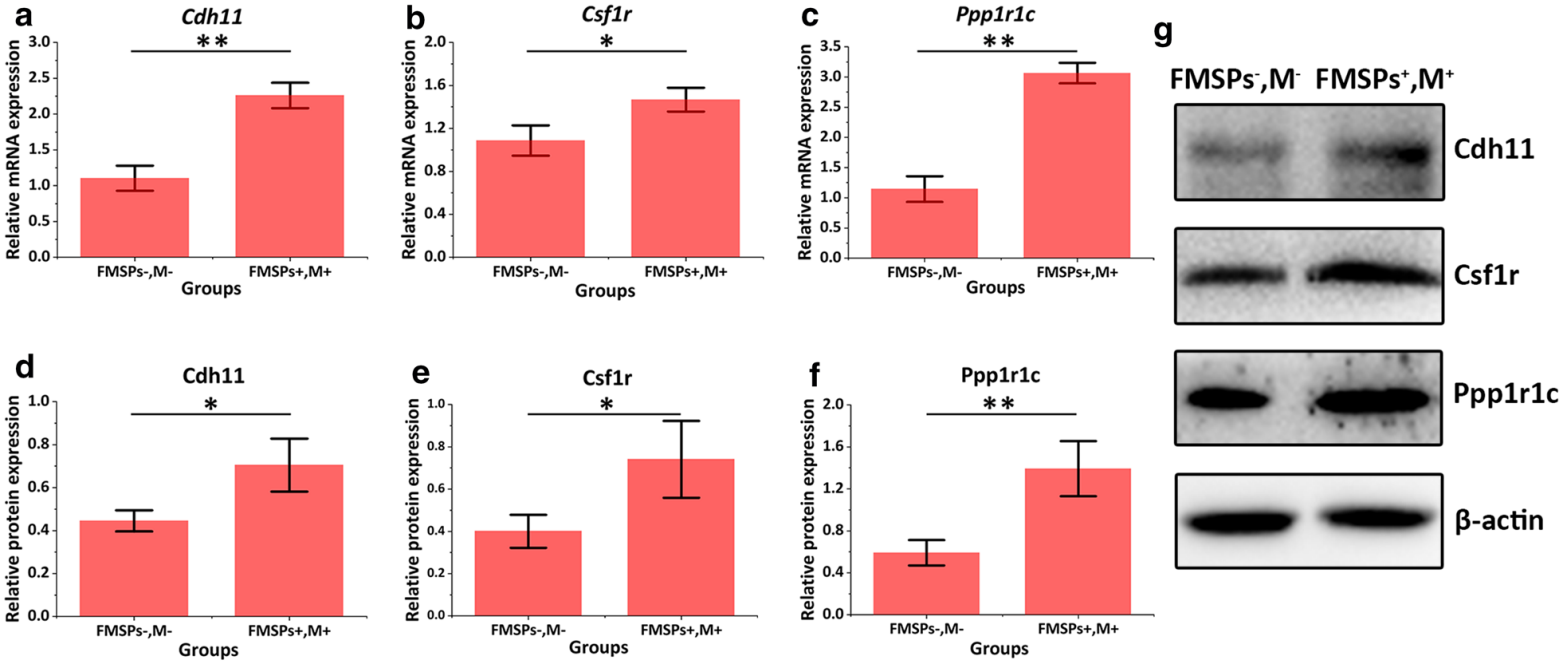
RT-qPCR and western blot verification of differentially expressed mRNAs. **a**–**c** RT-qPCR validation shows that the expression levels of *Cdh11*, *Csf1r* and *Ppp1r1c* were significantly upregulated in the treatment group compared with the blank control group. **d**–**g** Western blot validation showed that the protein levels of Cdh11, Csf1r, and Ppp1r1c were markedly increased in the treatment group compared with the blank control group. *P < 0.05, **P < 0.01

Previous studies have shown that axons will undergo rapid tensile growth under the action of external mechanical forces. When the axon length increases, the axon diameter also increases, accompanied by the synthesis of new microtubules and neurofilament proteins [51]. Because the application of forces to axons can induce rapid elongation without making the axon thinner [52–54], it can be said that the tensile growth of axons is not a viscoelastic deformation but substantial growth via continuous mechanical tension [55]. This implies that the stretching can be sensed by neurons to trigger additional protein synthesis and transport [56–59]. This process must involve the expression of certain key genes and the activation of signaling pathways. To further determine the effects of mechanical signals mediated by FMSPs on the gene expression profile in neural cells, mRNA transcriptome sequencing and bioinformatics analysis were performed on cell samples from different experimental groups. The mechanism by which magnetic mechanical force promotes axonal regeneration from the perspective of genetics is further expected to be elucidated. Since only the cooperation of FMSPs and magnetic fields can affect the regeneration of axons, cells from the treatment group (FMSPs^+^, M^+^) and the blank control group (FMSPs^−^, M^−^) were selected for gene sequencing. As shown in Fig. 8a, a volcano plot was used to assess gene expression variation between the treatment and blank control groups. After pairwise comparison, a total of 89 mRNAs displayed differential expression, including 43 upregulated mRNAs. The level of expression changes of differentially expressed mRNAs is exhibited in the heatmap (Fig. 8b). GO enrichment analysis was utilized to annotate genes and analyze the biological processes of genes. Figure 8c shows that a total of 43 upregulated mRNAs are mainly involved in the regulation of chemokine secretion process (GO: 0090197, 0090196, 0090195), central nervous system neuron differentiation and axonogenesis process (GO: 0021953, 0021952, 0021955), as well as regulation of mitotic cell cycle phase transition process (GO: 1900087, 1901990, 1902808, 1901987). Moreover, three target genes including *Csf1r*, *Cdh11* and *Ppp1r1c* were identified and screened among the biological processes highly correlated with axon growth from upregulated GO terms. The three identified differentially expressed mRNAs were further validated with reverse transcription-quantitative real-time PCR (RT-qPCR) analysis in PC12 cells. The expression levels of *Cdh11*, *Csf1r* and *Ppp1r1c* were significantly upregulated in the treatment group compared with the blank control group (P < 0.01) (Fig. 9a–c). Furthermore, the lysates from samples were subjected to western blotting. As shown in Fig. 9d–f, the protein levels of Cdh11, Csf1r, and Ppp1r1c were markedly increased in treated samples compared with the blank control sample (*P < 0.05, **P < 0.01). The findings further verify the accuracy of sequencing data and are consistent with the bioinformatics results.

The *Cdh11* gene encodes a type II classical cadherin (Cadherin-11) from the cadherin superfamily, integral membrane proteins that mediate calcium-dependent cell–cell adhesion. The expression of *Cdh11* is associated with growth cone movement during axonal migration [60]. Cadherin-11 is an axon growth-promoting factor that a previous study found to be involved in growing motor and sensory axons in the mouse embryo [61, 62]. In the regulation of axon growth, cadherin-11 plays an important role in adhesive interactions occurring between growing axons. Cadherin-11 promotes the fasciculation course of motor axon bundles [60] and the construction of neural networks [63] by regulating the interaction of growth cones for neighboring axons. The upregulated expression of the *Cdh11* gene and the increased translation of cadherin-11 protein in the treatment group are consistent with the observations of rapid and targeted axon growth, which suggests that mechanical forces mediated by FMSPs can promote *Cdh11* gene expression.

The *Csf1r* gene encodes the receptor for colony stimulating factor 1 (Csf1), a cytokine that controls the production, differentiation, and function of macrophages. This receptor mediates most of the biological effects of this cytokine. After a peripheral nerve lesion, the neuron undergoes a number of degenerative processes, so-called Wallerian degeneration, followed by attempts at regeneration. Breakdown of the axon distal to the site of injury is initiated 48 to 96 h after transection. The deterioration of myelin begins, and the axon becomes disorganized. Macrophages gathered around damaged areas, as immune cells, can phagocytose myelin and axonal debris [64], which is conducive to decreasing the inflammatory immune response [65–67] and promoting axonal regeneration by releasing a large number of regeneration-related factors, including extracellular matrix proteins, growth factors, cytokines and chemokines [32, 68–70]. Our results indicate that the combination of FMSPs and a magnetic field can improve the biological function of Csf1 cytokines by upregulating the expression of the *Csf1r* gene and in turn contribute to axonal regeneration through Csf1-dependent macrophage activation [71].

The *Ppp1r1c* gene encodes protein phosphatase 1 (PP1) regulatory inhibitor subunit 1C and belongs to a group of PP1 inhibitory subunits that are themselves regulated by phosphorylation. PP1 is a major serine/threonine phosphatase that regulates a variety of cellular functions and plays a critical role in the regulation of neuronal morphology. PP1 has been proven to promote axonal growth by the dephosphorylation of microtubule-associated proteins [72]. However, *Ppp1r1c*, as an upregulated gene, contributed to the development of axon growth in the current study. The possible reason for this seemingly contradictory effect may be the heterogeneity of samples between different experiments. There are few *Ppp1r1c* studies associated with axon growth. Thus, the potential molecular mechanism of *Ppp1r1c* for axon growth needs more evidence.

## Conclusion

In summary, we have successfully designed and prepared novel FMSPs for neural regeneration therapeutics. By virtue of their excellent biocompatibility and ability to interact with neural cells, the custom made FMSPs can be endocytosed into cells, transported along the axons, and then aggregated in the growth cones. Because of their high saturation magnetization, the mechanical forces generated by FMSPs can promote the growth and elongation of axons under external magnetic fields, enabling the precise and noninvasive remote manipulation of neuron regeneration. Furthermore, the mechanism by which magnetic mechanical force promotes axonal regeneration was studied by mRNA transcriptome sequencing and bioinformatics analysis. The results indicated that the combination of FMSPs and a magnetic field can improve the biological function of Cadherin-11 and Csf1 by upregulating the expression of *Cdh11* and *Csf1r* genes, which in turn contribute to axonal regeneration. Since the manipulation of neuronal growth is of great significance in the field of neural regeneration therapeutics, it is believed that our findings not only provide a powerful stimulator for axonal development but also explore the signaling pathway between mechanical force stimulation and the biological events of axon elongation.

## Supplementary information

**Additional file 1.** Additional Information.

**Additional file 2: Movie S1.** PC12 cells maintaining active mitotic proliferation and neurite outgrowth after the endocytosis of FMSPs. The arrows highlight the proliferating and differentiated cells.

**Additional file 3: Movie S2.** FMSP-treated DRG neurons showed neurite outgrowth and the formation of complex neuronal networks.

**Additional file 4: Movie S3.** FMSPs are transported along the axons and then aggregate in the growth cones.

**Additional file 5: Movie S4.** The neurites of PC12 cells treated with FMSPs tended to be arranged parallel to one another and grew preferentially along the direction of the magnetic field. The arrows highlight the neurites with directional growth.

**Additional file 6: Movie S5.** Dynamic growth process of DRG neuron axons in the treatment group (FMSPs^+^, M^+^).

**Additional file 7: Movie S6.** Dynamic growth process of DRG neuron axons in the FMSP control group (FMSPs^+^, M^−^).

**Additional file 8: Movie S7.** Dynamic growth process of DRG neuron axons in the magnetic field control group (FMSPs^−^, M^+^).

**Additional file 9: Movie S8.** Dynamic growth process of DRG neuron axons in the blank control group (FMSPs^−^, M^−^).

## Data Availability

All sequence data generated and analyzed during the current study are available in the NCBI database under the Project accession number PRJNA597946 (https://www.ncbi.nlm.nih.gov/sra/PRJNA597946).
