# Correction to: Growth and elongation of axons through mechanical tension mediated by fluorescent-magnetic bifunctional Fe_3_O_4_·Rhodamine 6G@PDA superparticles

**DOI:** 10.1186/s12951-021-00816-5

**Published:** 2021-05-17

**Authors:** Yang Wang, Binxi Li, Hao Xu, Shulin Du, Ting Liu, Jingyan Ren, Jiayi Zhang, Hao Zhang, Yi Liu, Laijin Lu

**Affiliations:** 1 Department of Hand Surgery, The First Hospital of Jilin University, Changchun, 130021 People’s Republic of China; 2 State Key Laboratory of Supramolecular Structure and Materials, College of Chemistry, Jilin University, Changchun, 130012 People’s Republic of China; 3 Institute of Translational Medicine, The First Hospital of Jilin University, Changchun, 130021 People’s Republic of China; 4 Departments of Geriatrics, The First Hospital of Jilin University, Changchun, 130021 People’s Republic of China

## Correction to: J Nanobiotechnol (2020) 18:64 https://doi.org/10.1186/s12951-020-00621-6

The authors regret errors in Fig. 2a, b, d and g in the originally published article [1]. In Fig. 2a, b and d, the TEM/HRTEM images and the magnetic curve of FMSPs were not the correct ones. These mistakes came from the confusion of the images and the curves. In Fig. 2g, the authors wish to use the different PL excitation and emission spectra of FMSPs to their another article. The authors apologize for these mistakes and are now providing the correct TEM/HRTEM images and magnetic curve, and the new PL excitation/emission spectra of FMSPs in Fig. 2. All the data herein are accurate and reproducible. These corrections do not affect the conclusion of this article.

**Figure:**
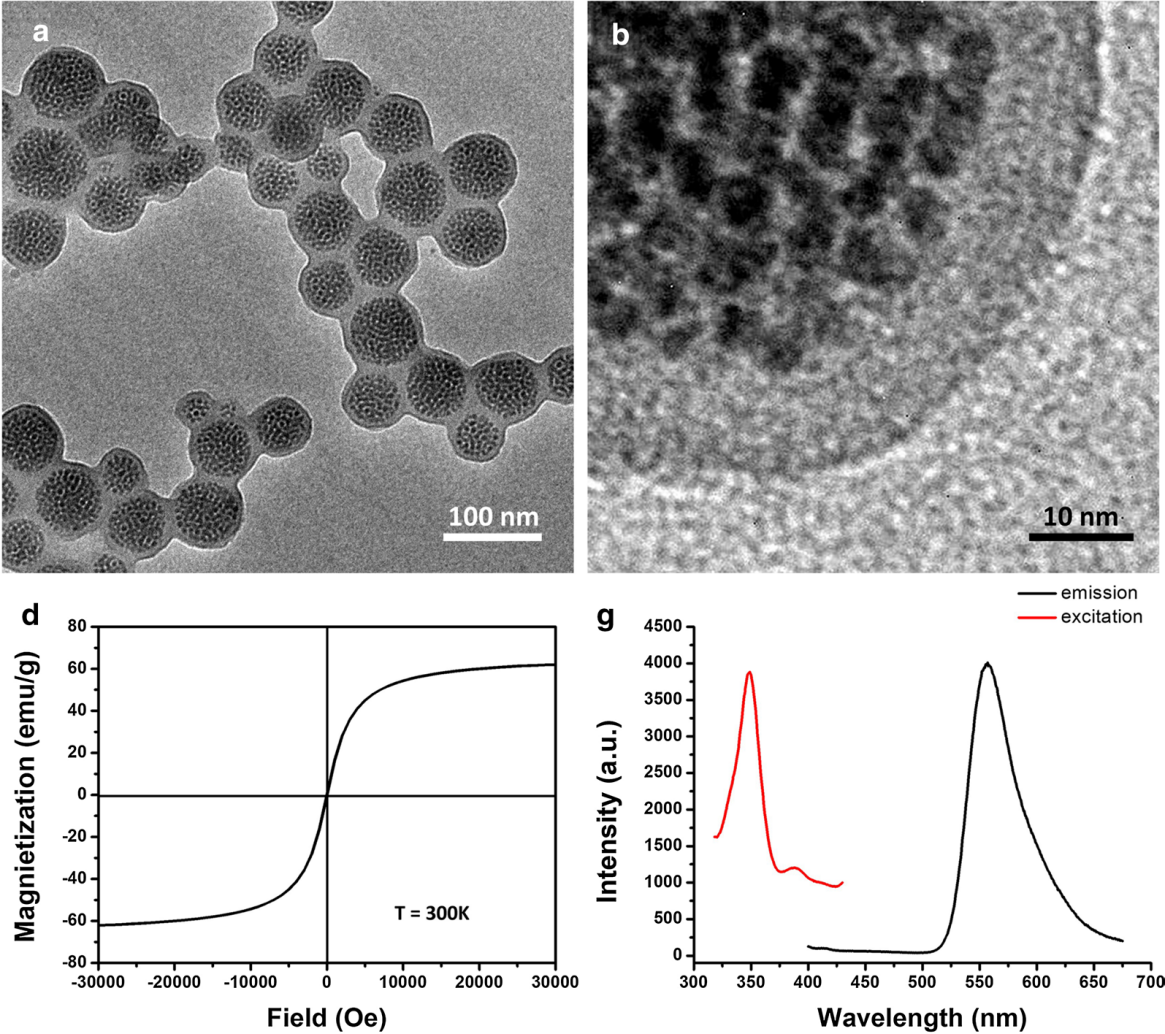

**Fig. 2** TEM (**a**) and HRTEM (**b**) images of FMSPs.** d** Magnetic curve of FMSPs.** g** PL emission and excitation spectra of FMSPs
