# Supplementary material for: Growth and elongation of axons through mechanical tension mediated by fluorescent-magnetic bifunctional Fe3O4·Rhodamine 6G@PDA superparticles
Source: J Nanobiotechnology. 2020 Apr 25;18:64. doi: 10.1186/s12951-020-00621-6 (PMC7183675; doi:10.1186/s12951-020-00621-6)

**Additional Information**

**Growth and Elongation of Axons through Mechanical Tension Mediated by Fluorescent-Magnetic Bifunctional Fe_3_O_4_·Rhodamine 6G@PDA Superparticles**

Yang Wang,^1^ Binxi Li,^2^ Hao Xu,^3^ Shulin Du,^2^ Ting Liu,^4^ Jingyan Ren,^1^

Jiayi Zhang,^1^ Hao Zhang,^2^ Yi Liu,^2*^ Laijin Lu^1*^

^1^Department of Hand Surgery, The First Hospital of Jilin University, Changchun, Jilin 130021, P. R. China

^2^State Key Laboratory of Supramolecular Structure and Materials, College of Chemistry, Jilin University, Changchun, Jilin 130012, P. R. China

^3^Institute of Translational Medicine, The First Hospital of Jilin University, Changchun, Jilin 130021, P. R. China

^4^Departments of Geriatrics, The First Hospital of Jilin University, Changchun, Jilin 130021, P. R. China

* Corresponding Authors: Laijin Lu: [lulaijin@hotmail.com](mailto:lulaijin@hotmail.com)

Yi Liu: yiliuchem@jlu.edu.cn

**Table of the Additional Information**

**1.** Extraction and culture of primary DRG neurons.

**2.** Specimen preparation of TEM.

**3**. Quality control of the RNA extraction and processing of raw sequencing data.

**4. Table S1.** The quality of the output of the sequencing data.

**5. Table S2.** Sequences of the primers used in this study.

**6. Fig. S1** Confocal laser scanning microscopy images of cells at a single focal plane verify the internalization of the FMSPs into the DRG neurons.

**7. Fig. S2** Concentration-dependent cellular uptake of FMSPs.

**8. Fig. S3** Time-dependent cellular uptake of FMSPs.

**9. Fig. S4** Energy-dependent cellular uptake of FMSPs.

**10. Fig. S5** Exocytosis of internalized FMSPs is studied using FACS.

**11. Fig. S6** Simulation and quantification of magnetic field.

**Extraction and culture of primary DRG neurons**

Primary DRG neurons were isolated from Sprague-Dawley rats (age 1-3 days). Dorsal ganglia of rat were dissected in Hank’s buffer solution and enzymatically treated in a solution of 2 ml trypsin (0.25%), 40μl collagenase (0.05%), 100 μl hyaluronidase (0.1%) and 2ml dispase II (1.25 U/ml) at 37 °C temperature for 30 min. The cells were additionally dissociated mechanically using injection needles and DMEM containing 10% FBS was used to stop the trypsinization process. Dissociated DRG neurons were centrifuged and collected at 1500 rpm for 5 min. Afterwards the cells were resuspended and maintained in neurobasal medium supplemented with 2% B27, 2 mM L-glutamine, 0.5% pen/strep and 50 ng/ml NGF. The cultures were conducted in an incubator at 37°C in a humidified atmosphere with 5% CO_2_.

**Specimen preparation of TEM**

For transmission electron microscopy (TEM), cells were washed three times with cold PBS, trypsinized, harvested, centrifuged, the supernatant was discarded and the pallet of fresh cells samples were fixed overnight with 2% glutaraldehyde solution. This was followed by secondary fixation with 1% osmium tetroxide for 2h. Then the samples were dehydrated in a graded series of ethanol ending with propylene oxide. Samples were then embedded in Epon resin. About 70 nm thin sections were prepared with a PowerTome-XL ultramicrotome (RMC, USA). The sections were observed using TEM operating at 120 kV.

**Quality control of the RNA extraction and processing of raw sequencing data**

The total RNA was respectively isolated from treatment and blank control group using TRIzol regents (Invitrogen, Carlsbad, CA, USA). Agarose gel electrophoresis was performed to determine the extent of RNA degradation and contamination, subsequently the purity was evaluated by spectrophotometry (Nanodrop, USA). RNA concentration was accurately quantified using Qubit fluorometer (Invitrogen, Carlsbad, CA, USA). In the following, the RNA integrity was detected by Agilent 2100 bioanalyzer (Agilent Technologies, CA, USA).

To ensure the accuracy of subsequent bioinformatics, low-quality reads (quality value﹤20) was trimmed, and the adaptor sequence, the reads with “N” > 10%, and the length of discard adapter or trimmed sequences < 20 bp were pruned away. A total of 46.28 G transcriptome clean data was obtained from 46.99 G raw data after filtering sequencing adapter sequence, low quality reads, high N% sequences and short sequences. As shown in Table S1, phread value > 20 was more than 98% in the samples. And then totals of 28763 mRNAs were acquired after comparison with rat reference.

**Table S1.** The quality of the output of the sequencing data

| Sample | Raw Base (G) | Clean Base (G) | Q20% | Q30% | GC% |
| --- | --- | --- | --- | --- | --- |
| Treatment group-1 | 6.87 | 6.78 | 98.34 | 95.02 | 48.77 |
| Treatment group-2 | 7.31 | 7.22 | 98.33 | 95.05 | 48.84 |
| Treatment group-3 | 7.91 | 7.80 | 98.35 | 95.12 | 48.94 |
| Blank control group-1 | 8.73 | 8.60 | 98.45 | 95.37 | 48.9 |
| Blank control group-2 | 7.85 | 7.70 | 98.14 | 94.58 | 49.53 |
| Blank control group-3 | 8.32 | 8.18 | 98.31 | 95.01 | 49.18 |

Q20, Q30%, the percentage of bases with Phred values greater than 20 and 30 in total. GC%, the sum of the amounts of bases G and C represents a percentage of the total base numbers.

**Table S2.** Sequences of the primers used in this study.

| Primer | Sense (5'-3') | Antisense (5'-3') |
| --- | --- | --- |
| Cdh11 | TCAGAACAGCCCTTCCCAATA | TCACCCTTCCTACTTCCTCCC |
| Csf1r | AAGGAAGGCCGAGGCTATG | GGTATCTACGCCCTGACTGGA |
| Ppp1r1c | AACTCCTCCTGGCCTATGGT | CAGAAGCATGCTAAATGCGGT |
| GAPDH | AGACAGCCGCATCTTCTTGT | CTTGCCGTGGGTAGAGTCAT |

**Fig. S1** Confocal laser scanning microscopy images of cells at a single focal plane verify the internalization of the FMSPs into the DRG neurons. (a) Tubulin immunostaining of cells. (b) Fluorescent FMSPs uptaken by the cells. (c) Nuclei were counterstained with blue-fluorescent DAPI. (d) Merged image. The images were captured at 40×obiective with 2.5×magnification.


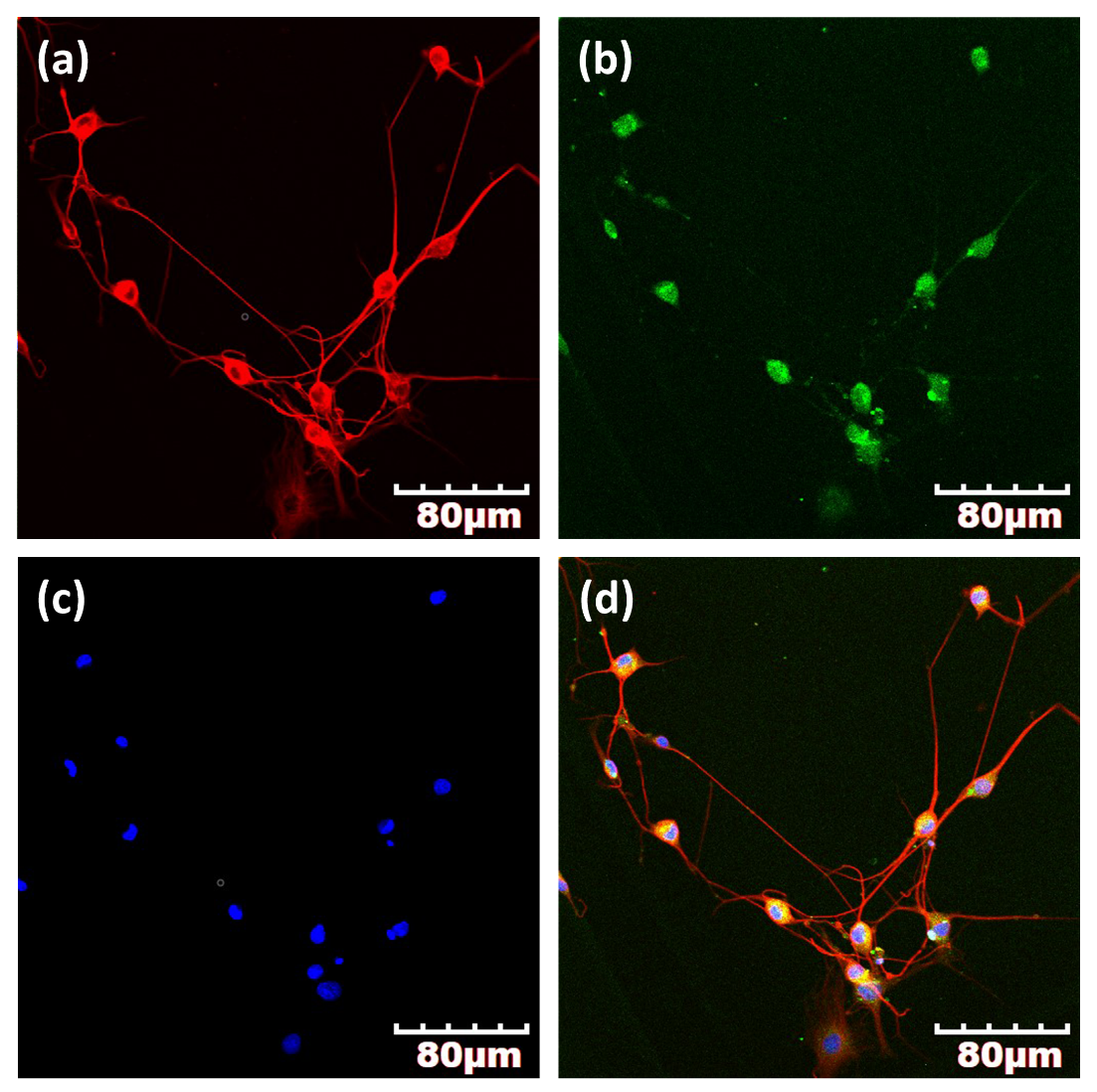


**Fig. S2** Concentration-dependent cellular uptake of FMSPs. (a-e) Fluorescence microscopy images of PC12 cells after incubation with different concentrations of FMSPs. (f) Intracellular fluorescent intensity measured by FACS. (g) Relationship between FMSPs uptake and concentrations. The images were captured at 200× magnification. Nuclei (blue) were stained with Hoechst nucleic acid stains.


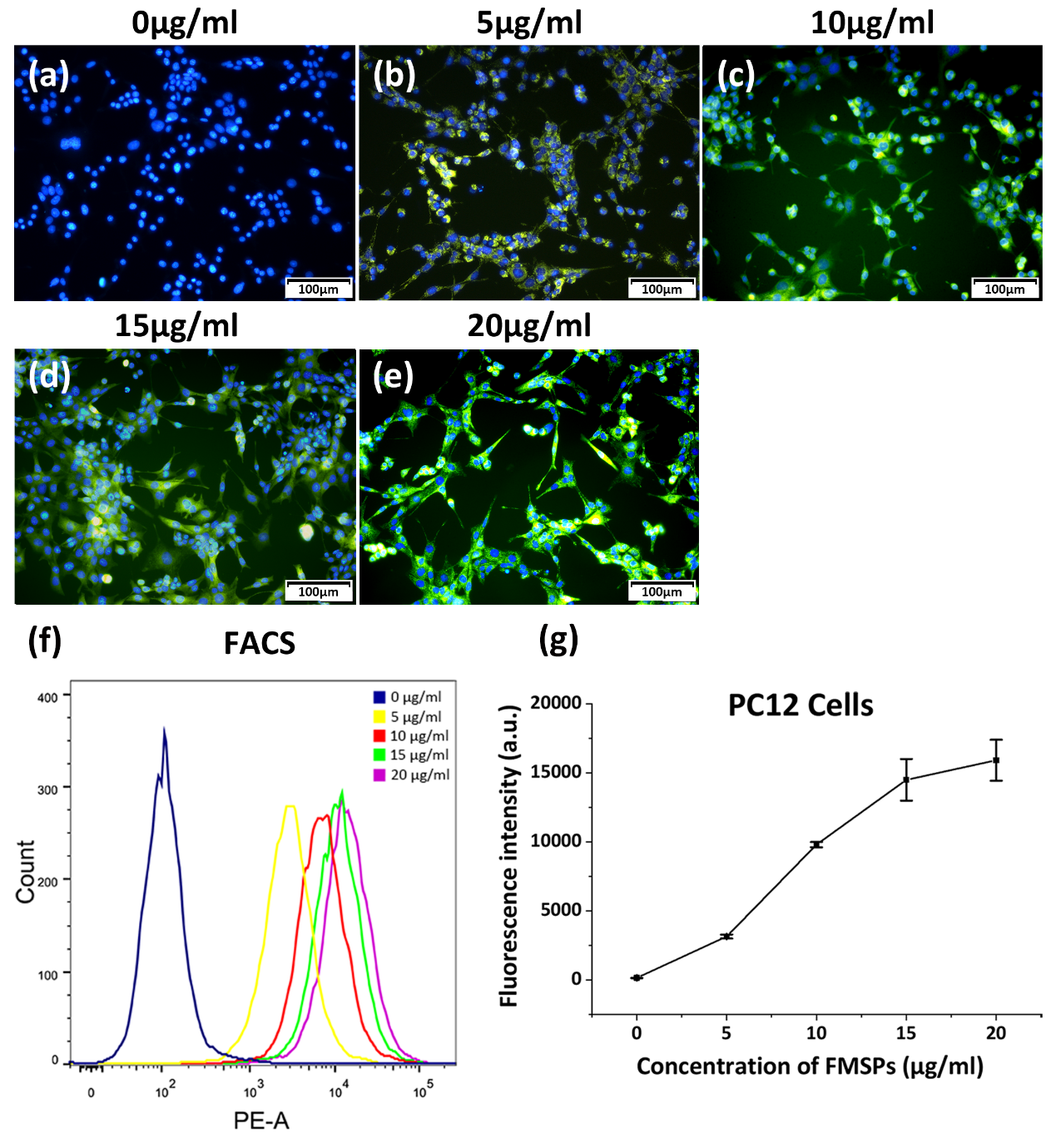


**Fig. S3** Time-dependent cellular uptake of FMSPs. (a-f) Fluorescence microscopy images of PC12 cells with different incubation durations. (g) Intracellular fluorescent intensity measured by FACS. (h) Relationship between FMSPs uptake efficiency and incubation time. The images were captured at 200× magnification. Nuclei (blue) were stained with Hoechst nucleic acid stains.


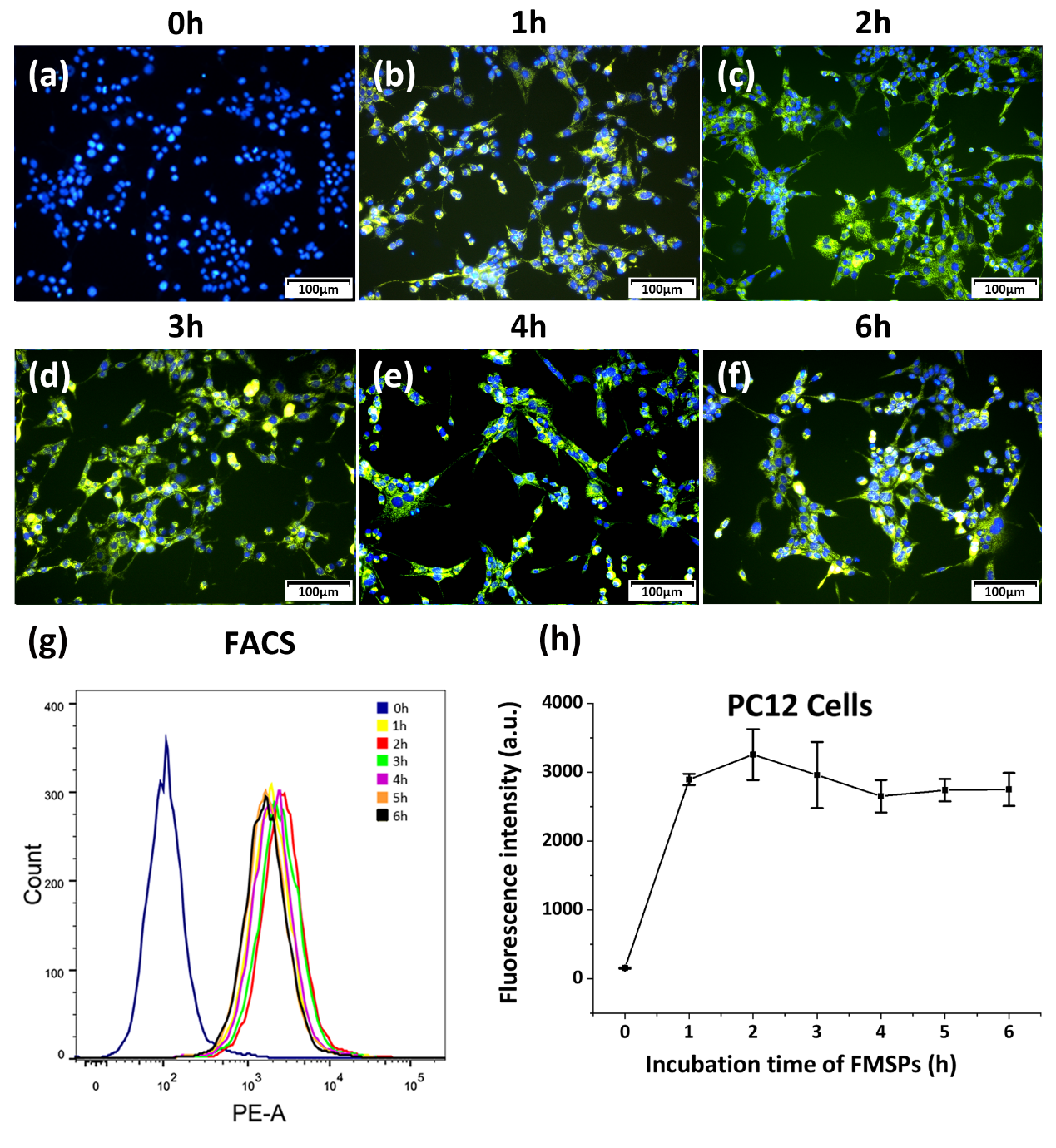


**Fig. S4** Energy-dependent cellular uptake of FMSPs. Fluorescence microscopy images of PC12 cells without treatment with FMSPs (a) and incubated with FMSPs (b). (c) Fluorescence microscopy images of PC12 cells incubated with FMSPs via 2-DG (c) and low temperature (d) treatments. (e,f) Intracellular fluorescent intensity measured by FACS. The images were captured at 200× magnification. Nuclei (blue) were stained with Hoechst nucleic acid stains.


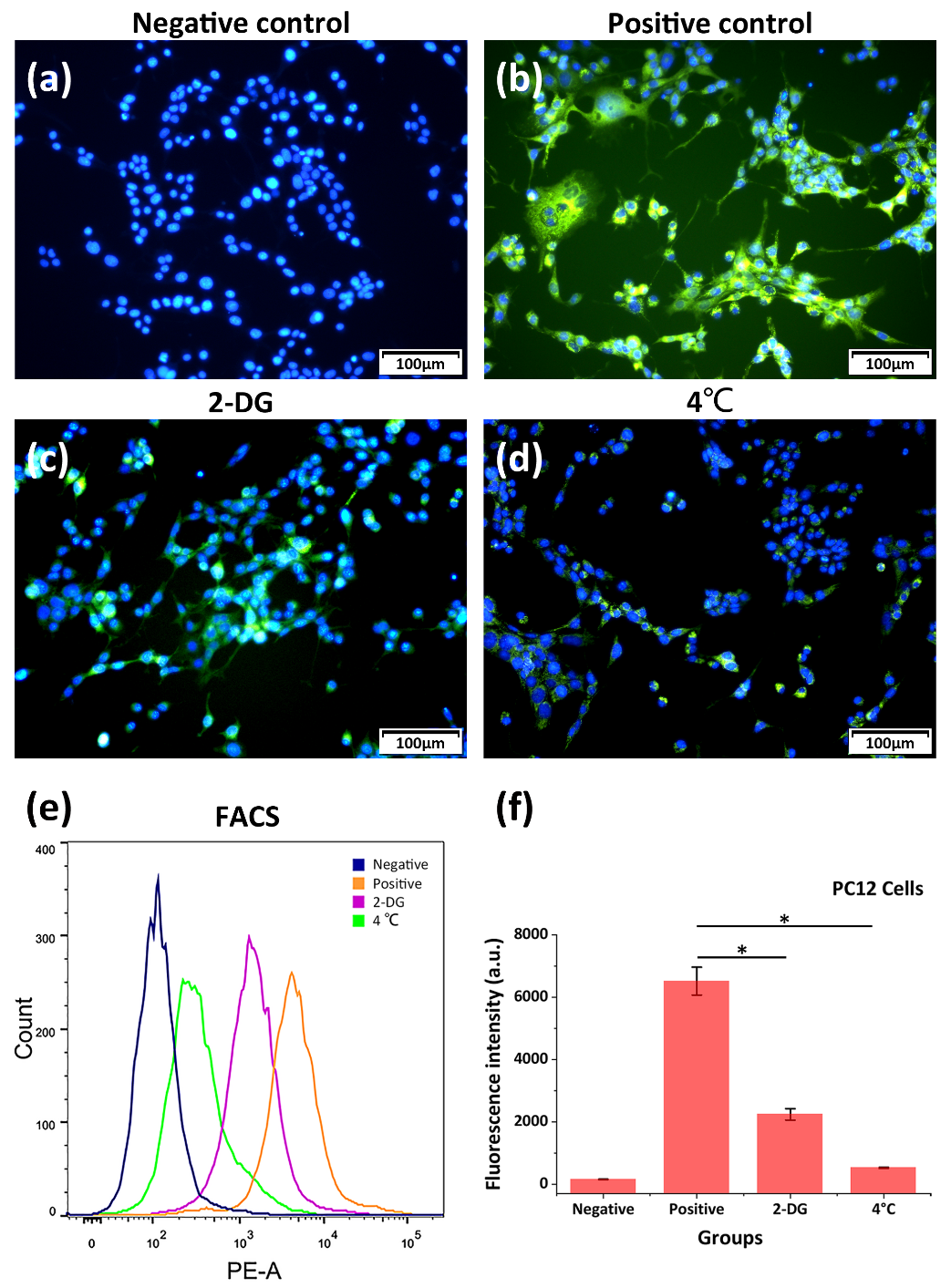


**Fig. S5** Exocytosis of internalized FMSPs is studied using FACS. (a-f) Cells morphology and status under different incubation time. (g) Intracellular fluorescence intensity of PC12 cells measured by FACS.


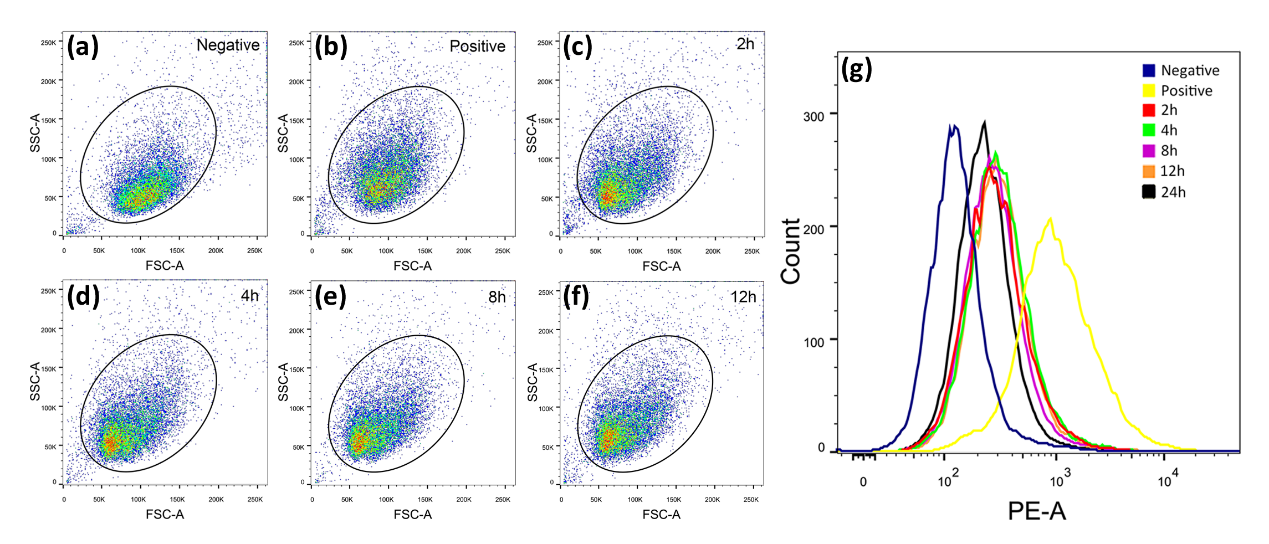


**Fig. S6** Simulation and quantification of magnetic field. (a) Distribution of magnetic field generated by one perpetual cuboid neodymium magnet. (b) Magnetic field strength to the cells at the center of the dish in the direction of the magnetic field gradient**.**


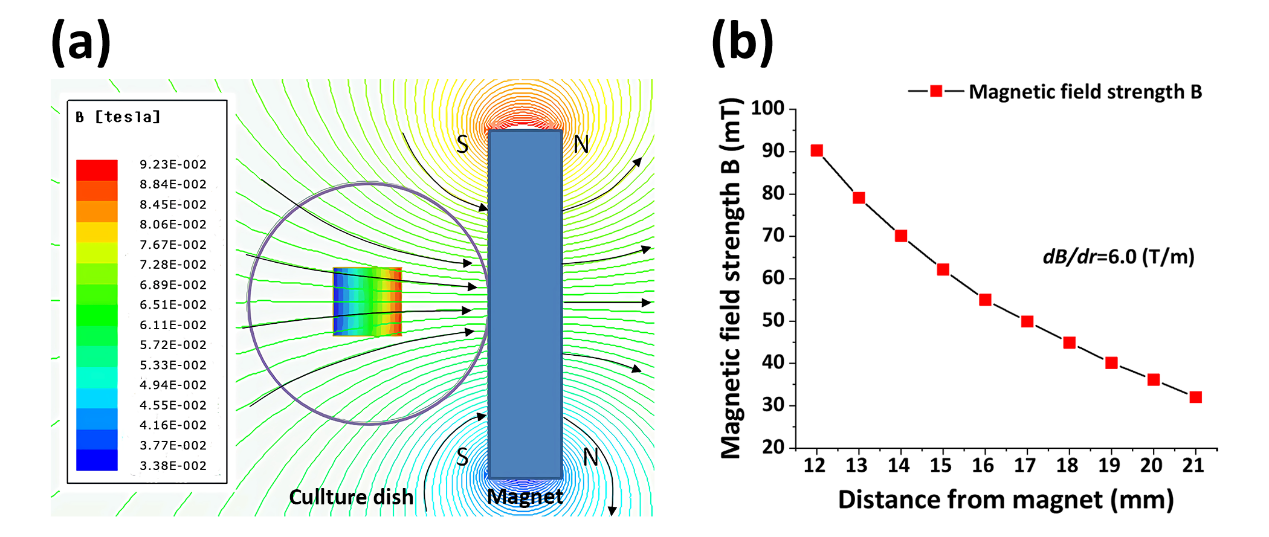

Supplement: Supplementary file 1 — Additional file 1. Additional Information. [file 12951_2020_621_MOESM1_ESM.docx]
